# Overcoming Clinical Inertia: A Randomized Clinical Trial of a Telehealth Remote Monitoring Intervention Using Paired Glucose Testing in Adults With Type 2 Diabetes

**DOI:** 10.2196/jmir.4112

**Published:** 2015-07-21

**Authors:** Deborah A Greenwood, Shelley A Blozis, Heather M Young, Thomas S Nesbitt, Charlene C Quinn

**Affiliations:** ^1^ Clinical Performance Improvement Consultant Office of Patient Experience, Quality and Clinical Effectiveness Sutter Health Sacramento, CA United States; ^2^ Program Coordinator Sutter Health Integrated Diabetes Education Network Sutter Medical Foundation Sacramento, CA United States; ^3^ Associate Professor Department of Psychology University of California Davis Davis, CA United States; ^4^ Associate Vice Chancellor for Nursing, Dean and Professor Betty Irene Moore School of Nursing University of California Davis Sacramento, CA United States; ^5^ Associate Vice Chancellor, Strategic Technologies and Alliances Director, Center for Health and Technology University of California Health System Sacramento, CA United States; ^6^ Associate Professor, Department of Epidemiology and Public Health School of Medicine Univeristy of Maryland Baltimore, MD United States

**Keywords:** telehealth, remote consultation, electronic health records, health records, personal, diabetes mellitus, type 2, self-care, monitoring, physiologic, blood glucose self-monitoring, hemoglobin A1c, glycosylated, eHealth, patient participation

## Abstract

**Background:**

Type 2 diabetes mellitus is a worldwide challenge. Practice guidelines promote structured self-monitoring of blood glucose (SMBG) for informing health care providers about glycemic control and providing patient feedback to increase knowledge, self-efficacy, and behavior change. Paired glucose testing—pairs of glucose results obtained before and after a meal or physical activity—is a method of structured SMBG. However, frequent access to glucose data to interpret values and recommend actions is challenging. A complete feedback loop—data collection and interpretation combined with feedback to modify treatment—has been associated with improved outcomes, yet there remains limited integration of SMBG feedback in diabetes management. Incorporating telehealth remote monitoring and asynchronous electronic health record (EHR) feedback from certified diabetes educators (CDEs)—specialists in glucose pattern management—employ the complete feedback loop to improve outcomes.

**Objective:**

The purpose of this study was to evaluate a telehealth remote monitoring intervention using paired glucose testing and asynchronous data analysis in adults with type 2 diabetes. The primary aim was change in glycated hemoglobin (A_1c_)—a measure of overall glucose management—between groups after 6 months. The secondary aims were change in self-reported Summary of Diabetes Self-Care Activities (SDSCA), Diabetes Empowerment Scale, and Diabetes Knowledge Test.

**Methods:**

A 2-group randomized clinical trial was conducted comparing usual care to telehealth remote monitoring with paired glucose testing and asynchronous virtual visits. Participants were aged 30-70 years, not using insulin with A_1c_ levels between 7.5% and 10.9% (58-96 mmol/mol). The telehealth remote monitoring tablet computer transmitted glucose data and facilitated a complete feedback loop to educate participants, analyze actionable glucose data, and provide feedback. Data from paired glucose testing were analyzed asynchronously using computer-assisted pattern analysis and were shared with patients via the EHR weekly. CDEs called participants monthly to discuss paired glucose testing trends and treatment changes. Separate mixed-effects models were used to analyze data.

**Results:**

Participants (N=90) were primarily white (64%, 56/87), mean age 58 (SD 11) years, mean body mass index 34.1 (SD 6.7) kg/m2, with diabetes for mean 8.2 (SD 5.4) years, and a mean A_1c_ of 8.3% (SD 1.1; 67 mmol/mol). Both groups lowered A_1c_ with an estimated average decrease of 0.70 percentage points in usual care group and 1.11 percentage points in the treatment group with a significant difference of 0.41 percentage points at 6 months (SE 0.08, t_159_=–2.87, *P*=.005). Change in medication (SE 0.21, t_157_=–3.37, *P*=.009) was significantly associated with lower A_1c_ level. The treatment group significantly improved on the SDSCA subscales carbohydrate spacing (*P*=.04), monitoring glucose (*P*=.001), and foot care (*P*=.02).

**Conclusions:**

An eHealth model incorporating a complete feedback loop with telehealth remote monitoring and paired glucose testing with asynchronous data analysis significantly improved A_1c_ levels compared to usual care.

**Trial Registration:**

Clinicaltrials.gov NCT01715649; https://www.clinicaltrials.gov/ct2/show/NCT01715649 (Archived by WebCite at http://www.webcitation.org/6ZinLl8D0).

## Introduction

In the United States, 9.3% of Americans have diabetes mellitus; of those, 90% to 95% are diagnosed with type 2 diabetes [1]. When uncontrolled, diabetes is the seventh leading cause of death and the leading cause of kidney failure, blindness, and nontraumatic amputations in the United States [1]. Achieving national diabetes outcome targets for blood glucose, blood pressure, and blood fats can decrease complications and improve quality of life [2]. However, research indicates people with diabetes remain at suboptimal glucose control for 2.9 years from patient and provider clinical inertia limiting treatment intensification [3,4]. Self-management of diabetes is a critical component of diabetes care [2] and self-monitoring of blood glucose (SMBG) is an essential self-management behavior [2,5]. Evaluation of SMBG data by primary care providers encourages more frequent medication changes and several studies indicate improved glycemic control [6-8]. Practice guidelines promote the use of SMBG for informing health care providers about glycemic control and providing patient feedback to increase knowledge, self-efficacy, and behavior change [9-11]. Effective SMBG includes structured behaviors such as (1) frequency of glucose testing, (2) participant use and response to glucose data, (3) health care provider data interpretation, and (4) therapy modifications [12,13]. However, there is controversy regarding the benefit of SMBG to improve glycated hemoglobin (A_1c_)—a measure of overall blood glucose control—in persons with noninsulin-treated type 2 diabetes with some systematic reviews reporting no reduction in A_1c_ [14,15]. However, current research incorporating structured monitoring profiles—defining the frequency, intensity, and timing of SMBG—shows significant improvement in A_1c_ [6,7,16,17]. Although there is limited research to document the most effective SMBG profile, paired glucose testing (eg, pairs of glucose results obtained before and after a meal, physical activity, or other event) is one suggested profile [10,18]. However, it is challenging to access glucose data frequently to interpret values and recommend patient actions.

A complete feedback loop—data collection and interpretation combined with feedback to the patient to modify treatment plan—has been associated with improved outcomes [19]. Although the complete feedback loop is an essential component of both SMBG [20] and remote monitoring, there is limited and inconsistent incorporation of SMBG feedback in diabetes management [14,19-21]. Although research has evaluated telehealth remote monitoring glucose data, few studies have incorporated SMBG profiles that provide timely feedback to patients and allow for real-time decision making [22].

**Figure 1:**
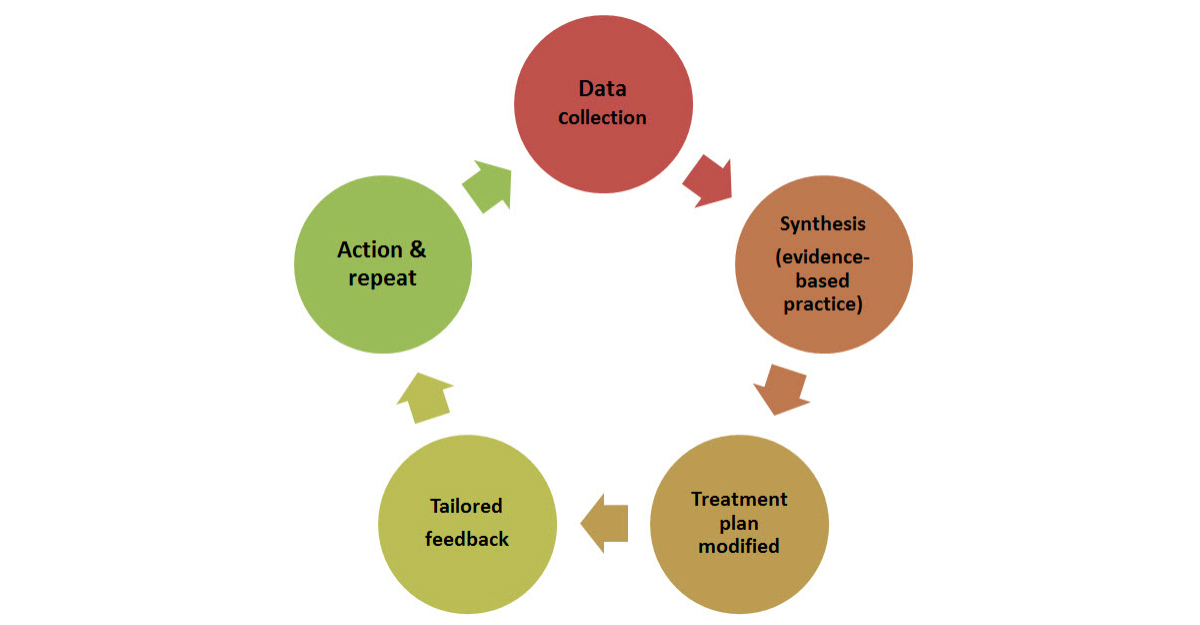
Complete feedback loop for improved outcomes in diabetes management.

In primary care, health care providers are often not prepared to interpret SMBG data, respond to patterns, and implement a complete feedback loop with tailored feedback for behavior change or treatment modifications [14,23]. Diabetes management programs with nurse care coordination [14,23] often include diabetes education provided by certified diabetes educators (CDEs), who are uniquely qualified to analyze SMBG data and problem solve with patients. Incorporating telehealth remote monitoring with CDE support employs the complete feedback loop to improve outcomes. Figure 1 shows the complete feedback loop elements [19]. The patient generates glucose data following targeted education on the elements of structured SMBG. Next, data are analyzed and synthesized by both the CDE and the patient using pattern management and evidence-based guidelines. In collaboration, the CDE and patient agree on modification of the existing treatment plan through active communication and tailored feedback from the CDE. Finally, a new action plan is developed using shared decision making and implemented by the patient and the cycle continues.

Telehealth remote monitoring may improve clinical outcomes, care coordination, engagement, and satisfaction [24,25]. Novel clinical interventions are needed that expand existing paradigms of diabetes care by utilizing telehealth remote monitoring and actionable patient-generated data for timely behavior and treatment changes. The purpose of this study was to test the effectiveness of a telehealth remote monitoring intervention with paired glucose testing for adults with noninsulin-treated type 2 diabetes. The hypothesis was that the intervention would result in a greater change in A_1c_ and improved self-management, self-efficacy, and diabetes knowledge compared to usual care over 6 months.

## Methods

### Study Design

The study was a 2-group randomized clinical trial with 1:1 randomization to usual care or telehealth remote monitoring with paired glucose testing (treatment group). Sample size was determined based on the main outcome: mean change in A_1c_ between treatment and usual care over 6 months. The comparison of usual care (n=39) to treatment participants (n*=*39) had 80% power to detect a 0.9% difference in A_1c_ between treatment and usual care after 6 months (α=.05, 2-tailed). A 15% additional margin for participant dropout resulted in a sample size of n=45 per group.

### Setting

The study was conducted between January and October 2013 in a large health care system in California with an established diabetes management program with telephonic nurse care coordination for diabetes population health management. CDEs proactively telephoned patients with A_1c_ ≥10% (86 mmol/mol) to develop care plans, whereas patients in lower risk groups (A_1c_ 7.5%-9% or 58-75 mmol/mol) called the program if desired. CDEs were trained in motivational interviewing to support behavior change, structured paired glucose testing, pattern management, and medication management. Approximately 7000 patients were enrolled in the diabetes management program at that time and nearly 1500 patients met the inclusion criteria.

### Recruitment and Enrollment

Participants were recruited through query of the electronic health record (EHR) and diabetes management database using the *International Classification of Diseases, Ninth Revision* (ICD-9) code 250.02. The inclusion criteria were:

1. Type 2 diabetes diagnosis treated with oral antihyperglycemic medications, noninsulin injectable medications, or lifestyle alone;
2. Participant in the diabetes management program for previous 12 months;
3. Aged between 30 and 70 years;
4. A_1c_ between 7.5% and 10.9% (58-96 mmol/mol) in previous 6 months;
5. Internet or 3G connection with email access;
6. Landline or cellular phone;
7. English speaking; and
8. Primary care provider in health system.

Exclusion criteria identified by medical chart review included:

1. Insulin prescription;
2. Unable to independently self-manage (diagnosis of dementia, severe depression, schizophrenia, or cognitive impairment for previous 12 months); and/or
3. Diagnoses of debilitating stroke, heart failure, end-stage renal disease, or legally blind.

**Figure 2:**
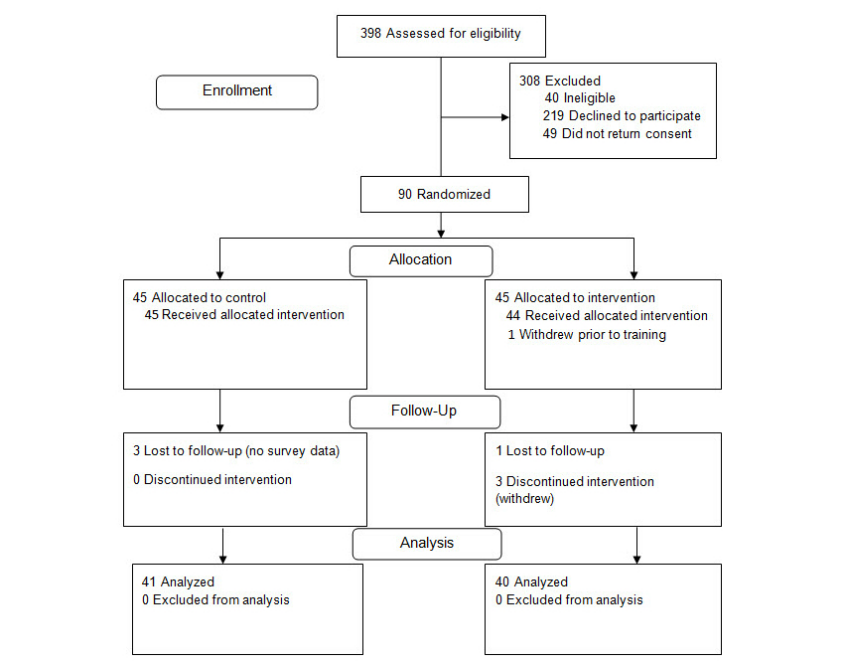
CONSORT flowchart of enrollment and participant status.

Eligible participants were contacted through mail, email, and telephone (see Figure 2). Consent forms were mailed and emailed to participants. The research team obtained informed consent by telephone and then participants signed consents and returned by postal mail. We estimated a 15% enrollment rate, but 6% was attained. Major reasons for ineligibility were non-English speakers (21%, 8/37), insulin (21%, 8/37), primary care provider not in health system (16%, 6/37), and no Internet access (14%, 5/37).

A permuted block, with blocks of 4 and 6, and a computer-generated random number table were utilized for randomization. After participants signed the consent form, the research coordinator assigned sequential study identification (ID) numbers. The investigator matched the ID numbers to the random number table to assign study group. Participants were notified of group assignment by email after completing online baseline self-assessment questionnaires. Participants randomized to the control group (usual care) were informed to continue in the diabetes management program for usual care. Blinding of participants, providers, and the research team was not possible.

Participants in both groups received a US $10 gift card after completing online questionnaires. A_1c_ tests were ordered every 3 months, as is standard of care when A_1c_ is elevated, then billed to insurance. A_1c_ tests were collected at health system laboratories using similar equipment following standardized procedures. Questionnaires were completed online using the Research Electronic Data Capture (REDCap) database. The study was approved by Sutter Health Central Institutional Review Committee and a Data and Safety Monitoring Board (DSMB) reviewed the study procedures and adverse events.

### Measures

#### Primary Outcome

The primary outcome was the difference in mean change in A_1c_ from baseline to 6 months between groups. A_1c_ at recruitment (prestudy A_1c_), baseline, and 3 and 6 months postprogram was obtained from EHR review. Baseline data were the most recent A_1c_ recorded before study enrollment in the previous 6 months. At the conclusion of the study, the A_1c_ was scheduled within a 3-month time period, approximately 4 weeks before and 8 weeks after the 6-month due date.

#### Secondary Outcomes

##### Diabetes Knowledge

Diabetes knowledge was measured using the Diabetes Knowledge Test (DKT) [26], a valid and reliable measure for estimating general understanding of diabetes, including healthy eating and glucose monitoring consisting of 23 multiple-choice items. The first 14 items, appropriate for people not using insulin, were administered to study participants. Scores are measured as the number of correct answers divided by the possible total of 14.

##### Self-Management

Self-management was measured by the Summary of Diabetes Self-Care Activities (SDSCA) [27], a 12-item self-report questionnaire with subscales: general diet, specific diet, carbohydrate spacing, exercise, monitoring blood glucose, and foot care. For example, participants were asked: “In how many of the past 7 days (0-7) did you check your blood glucose?” Higher scores indicate better self-care behavior.

##### Self-Efficacy

Self-efficacy was measured by the Diabetes Empowerment Scale short form (DES-SF), an 8-item measure of psychosocial self-efficacy in people with diabetes [28]. Scores ranged from 1-5, with 5 indicating “strongly agree.” The mean score of 8 items is reported.

### Usual Care/Control Group

Participants in usual care received diabetes education booklets and referral for formal diabetes education as needed. This group continued to receive nurse care coordination including reminders for A_1c_ and health maintenance exams sent by postal mail. The CDEs evaluated SMBG data when reported by participants (no specific monitoring profile was required) and discussed behavior changes with participants by telephone and/or secure messaging and discussed possible medication changes with their primary care provider through the EHR staff messaging tool. CDE contact with the usual care group was documented in the study database.

### Treatment Group: Structured Glucose Monitoring

The intervention incorporated a complete feedback loop and all essential elements of structured monitoring. Before the intervention, 6 CDEs attended in-person training sessions on intervention procedures, paired testing, and the goal of implementing a complete feedback loop. Participants in the treatment group attended a 1-hour, small group, in-person training session led by the CDE that included (1) use of the glucose meter, (2) implementation of the complete feedback loop, (3) use of paired glucose testing (frequency and intensity of monitoring), (4) American Diabetes Association (ADA) goals for pre- and postmeal, (5) how to use SMBG data to modify behavior or treatment, (6) expected feedback from CDEs with communication by secure message or phone, and (7) the use of shared decision making to implement the treatment plan [22]. Participants created a “personal experiment” and agreed to check glucose before and 2 hours after the same meal or physical activity for 1 week, and created a behavior change action plan to evaluate changes in SMBG data.

During training, participants were educated on how to use the Care Innovations Guide, a telehealth remote monitoring system approved by US Food and Drug Administration (FDA), which includes an in-home tablet computer connected by Internet or 3G network to the Care Innovations Health Suite online portal (Intel-GE Care Innovations, Roseville, CA, USA). The Care Innovations Guide is connected to the glucometer via USB cables and has a touchscreen for participants to answer daily health session questions. Data are downloaded to the Health Suite for CDEs to access via the Internet. Participants received a OneTouch Ultra 2 glucometer (approved by the FDA), test supplies, and USB cables to keep (Johnson and Johnson-Lifescan Inc, Milpitas, CA, USA) at no charge. Participants returned the Care Innovations Guide when the intervention concluded.

The 84 sequential daily health sessions, designed by the research team, were delivered electronically through the Care Innovations Guide as a text document in the style of a PowerPoint slide or via short video clips. The daily health session started with an audible prompt from the Care Innovations Guide at a time convenient for the participant, then participants completed a glucose check while the glucometer was connected to the Care Innovations Guide via the USB cable. Glucose data were automatically transferred to the Care Innovations Guide at that time. Participants read brief educational content focusing on 1 or 2 key points from the American Association of Diabetes Educators AADE7 [5] self-care behaviors (healthy eating, being active, monitoring, taking medication, problem solving, reducing risks, and healthy coping) a framework for organizing education and structuring behavior change goals. An automated health session reminded participants to evaluate glucose data, using pattern management, every week and to revise or continue their personal experiment for the following week. Participants were assigned a CDE to contact by secure message or phone for diabetes-related questions, instructed to contact their primary care provider for additional health care needs, and to call 911 for emergencies.

### Data Review and Nurse Care Coordination

**Figure 3:**
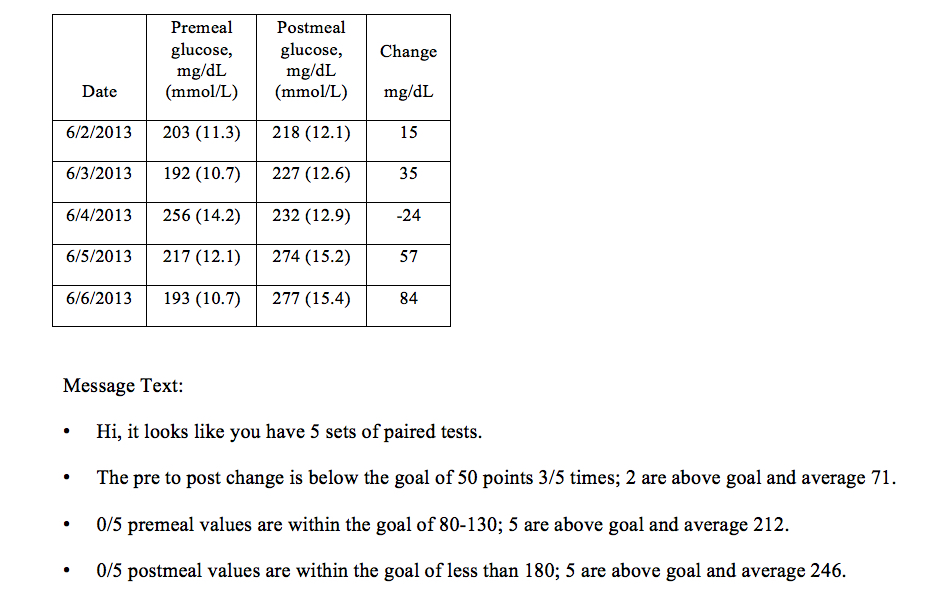
Sample weekly paired glucose testing data analysis, by software designed for the study, and sample message text for feedback to participants through asynchronous secure messaging via the electronic health record.

The CDEs reviewed health session and SMBG data in the Health Suite Web portal, stratified by a stoplight system with red indicating missing data or data above or below predetermined thresholds, yellow indicating pending data, and green indicating all data within range. CDEs telephoned participants, at predetermined times, when SMBG data indicated an urgent situation, such as severe hypoglycemia (1 value <50 mg/dL) or hyperglycemia (1 value >450 mg/dL). CDEs also telephoned participants if they reported a change in their feet or a new problem with medication by answering “yes” to health session questions. The Web portal data were reviewed by CDEs during normal business hours Monday through Friday. Data entered during nonbusiness hours were reviewed the following business day. Glucose data were analyzed weekly via software specifically designed for the intervention and evaluated against ADA goals of 80-130 mg/dL before meals, ≤180 mg/dL 2 hours postmeal, and a 30-50 point change between premeal to postmeal. After SMBG analysis, CDEs generated a virtual visit via asynchronous secure messaging through the EHR using the secure message feature. CDEs created a virtual encounter in the EHR, then “copy and pasted” a summary of SMBG pattern analysis data along with personalized feedback and individualized care coordination to reinforce action plans to create the virtual visit for both participants and providers to read (Figure 3). CDEs telephoned participants at weeks 4, 8, and 12 for a 30-minute discussion of SMBG trends, patterns, and goal achievement using motivational interviewing to identify opportunities to improve glucose values. If SMBG data did not improve after 4 weeks, CDEs discussed medication options with patients and/or primary care providers using shared decision making [29]. CDEs incorporated virtual visit data, both preprandial and postprandial glucose, to suggest medication changes, including insulin therapy. Medication changes were ordered by primary care providers via the EHR. Participants were instructed to use paired glucose testing or a monthly, 3-day 7-point glucose profile until the 6-month A_1c_. CDEs documented patient contact in the study database.

### Statistical Analysis

Mixed-effects models were used to compare mean change over time in primary and secondary outcomes between groups. A_1c_ was measured at baseline and at approximately 3 and 6 months. Change in A_1c_ was evaluated by fitting different growth models. Time was represented in the models by 90-day increments with the estimated change in A_1c_ equaling the amount of change of approximately 3 months. An indicator of group membership was added to the best-fitting growth model to test for differences between groups in A_1c_ at 3 and 6 months and the change in A_1c_ statistically adjusting for prestudy A_1c_. An indicator variable was added to the model that denoted whether a participant changed medication during the study and tested the effect of medication change on A_1c_ at 3 and 6 months, and on the change in A_1c_. Finally, a model was fit to test the effect of the number of paired glucose tests on A_1c_ at 3 and 6 months and change in A_1c_ over time controlling for change in medication and prestudy A_1c_. The effect of the number of paired glucose tests on A_1c_, accounting for effects due to changes in medication, was studied. Tests used a significance level of *P*<.05 or a 95% confidence interval that excluded zero. SAS version 9.4 (SAS Institute, Inc, Cary, NC, USA) was used to obtain restricted maximum-likelihood estimates using PROC MIXED.

## Results

### Overview

Table 1 shows baseline characteristics of participants (N=90). The majority were white (64%, 56/87), had diabetes for a mean 8.2 (SD 5.4) years, mean age of 58 (SD 11) years, and mean A_1c_ of 8.3% (SD 1.1; 67 mmol/mol). Participants were highly educated (63%, 55/87 college/postcollege), employed (53%, 46/87), with previous diabetes education (86%, 75/87), hypertension (59%, 51/87), and hyperlipidemia (69%, 60/87). There were no differences between groups at baseline except for self-reported hyperlipidemia (*P*=.006) in the treatment group. All data were included in an intent-to-treat analysis. There were no serious hyper- or hypoglycemic events or hospitalizations. One participant visited the emergency department unrelated to the study and DSMB determined there were no serious adverse events related to the study.

**Table 1:** Demographic and key baseline characteristics by group.

Characteristic		Usual care n=45	Treatment n=45
Female, n (%)		19 (21)	23 (25)
Age (years), mean (SD)		57.5 (10.6)	53.9 (10.4)
Years with diabetes (years),^a^ mean (SD)		8.1 (5.3)	8.3 (5.5)
**Ethnicity^a^, n (%)**			
	Hispanic	8 (9)	6 (7)
	White	27 (31)	29 (33)
	Black/African American	2 (2)	1 (1)
	American Indian	1 (1)	2 (2)
	Asian/Pacific Islander	4 (5)	3 (3)
	Other	1 (1)	2 (2)
	Not reported	0 (0)	1 (1)
**Education^a^, n (%)**			
	College	18 (21)	17 (20)
	High school	10 (12)	16 (18)
	Other	3 (3)	3 (3)
	Post college	12 (14)	8 (9)
**Employment status^a^, n (%)**			
	Employed	21 (24)	25 (29)
	Not employed	6 (7)	6 (7)
	Retired	16 (18)	13 (15)
**Marital status^a^, n (%)**			
	Married	29 (33)	36 (41)
	Single/divorced/widowed	14 (16)	8 (9)
Previous diabetes education^a^, n (%)		40 (46)	35 (40)
Computer use^a^, n (%)		42 (48)	44 (51)
**Type of Internet use^a^, n (%)**			
	Email, yesterday	34 (39)	41 (47)
	News, yesterday	24 (28)	34 (39)
	Medical, yesterday	9 (10)	14 (16)
	Video, yesterday	11 (13)	14 (16)
**Treatment^a^, n (%)**			
	Lifestyle	2 (2)	3 (3)
	Pills	10 (12)	7 (8)
	Pills and lifestyle	27 (31)	30 (35)
	Noninsulin injectable	2 (2)	3 (3)
	Noninsulin injectable and pills	2 (2)	1 (1)
**Comorbidities^a^, n (%)**			
	Heart attack	6 (7)	5 (6)
	Coronary heart disease	5 (6)	4 (5)
	Atherosclerosis	3 (3)	2 (2)
	Stroke	3 (3)	0 (0)
	Hypertension	22 (25)	29 (33)
	High cholesterol	24 (28)	36 (41)^b^
**Satisfaction with care^a^, n (%)**			
	Strongly agree	11 (13)	6 (7)
	Somewhat agree	14 (16)	13 (15)
	Neutral	9 (10)	12 (14)
	Somewhat disagree	8 (9)	6 (7)
	Strongly disagree	1 (1)	7 (8)
Body mass index (kg/m^2^), mean (SD)		34.1 (6.6)	34.1 (6.8)
**Blood pressure (mm Hg), mean (SD)**			
	Systolic	128.8 (13.9)	126.9 (13.2)
	Diastolic	76.6 (11.0)	77.3 (9.1)
Triglycerides (mg/dL), mean (SD)		175.5 (111.3)	170.5 (112.3)
High-density lipoprotein (mg/dL), mean (SD)		39.8 (10.6)	37.9 (12.2)
Low-density lipoprotein (mg/dL), mean (SD)		92.1 (29.4)	92.8 (28.8)
Cholesterol (mg/dL), mean (SD)		164.4 (35.6)	161 (38)
**A_1c_, mean (SD)**			
	%	8.2 (1.1)	8.5 (1.1)
	mmol/mol	7 (13)	69 (12)

^a^ Missing baseline questionnaire data for 3 participants (n=87).

^b^ *P*=.006; 2-tailed *P* value corresponding to a test of a difference between usual care and treatment groups.

### Primary Outcomes

The best-fitting model to describe A_1c_ over time was a quadratic growth model suggesting that the rate of change in A_1c_ was not constant over time. The quadratic model included 3 coefficients. The first two, the intercept and the linear change rate, described A_1c_ level and change in A_1c_ at specific time points. We evaluated these 2 coefficients at baseline and at 3- and 6-months postbaseline. Time was coded to reflect change at approximately 3-month intervals; thus, the linear change rate was the expected rate of change using a 90-day interval. The third coefficient, the quadratic effect, was the acceleration rate that allowed for a nonconstant rate of change over time. Of the 3 coefficients, only the intercept varied across individuals suggesting individual differences in A_1c_ levels over time, but no significant individual differences in the change rate. After fitting a growth model to data, individual scores may correlate between measurements. Model fit for A_1c_ scores was improved by allowing the residuals between measurements to correlate within persons.

**Figure 4:**
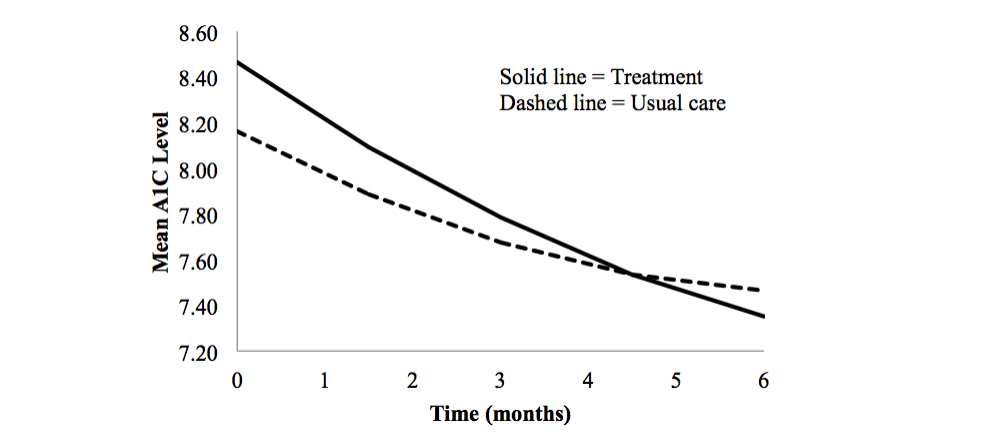
Estimated A1C trajectories for the usual care and treatment groups from baseline to 6 months.

Using a quadratic growth model to describe A_1c_ over time, we tested group differences in A_1c_ levels and the linear rates of change at baseline and at 3 and 6 months controlling for prestudy A_1c_. Comparisons between usual care group and treatment group suggested no difference in mean A_1c_ or in the linear change rate at baseline. An estimated group mean A_1c_ difference of 0.11 (*t*_159_=0.63, *P*=.53) at 3 months and –0.11 (*t*_159_=–0.59, *P*=.55) at 6 months showed no significant differences between groups. At 3 months, the usual care group decreased A_1c_ at a mean rate of –0.35 units (*t*_159_=–4.37, *P*<.001). Between groups, the difference in the change rate of –0.21 (*t*_159_=–1.87, *P*=.06) was not significant, suggesting no difference in the change rate at 3 months. At 6 months, the change rate in A_1c_ for the usual care group of –0.07 (*t*_159_=–1.41, *P*=.16) was not statistically significant, indicating no further improvement in A_1c_ at 6 months. However, the groups differed significantly in the change rate at 6 months, with the treatment group decreasing 0.23 units faster than the usual care group (*t*_159_=–2.87, *P*=.005) (Table 2). Figure 4 shows A_1c_ trajectories for groups over 6 months. Finally, the estimated acceleration rate for the usual care group was 0.14 (*t*_159_=4.07, *P*<.001), suggesting that the change rate increased with time. The estimated acceleration rate for the treatment group was 0.14 + (–0.013)=0.13, although the difference in this coefficient between groups was not significant (*t*_159_=–0.26, *P*=.80) suggesting no difference in this aspect of change in A_1c_ over time.

**Table 2:** Estimated mean A_1c_ level and instantaneous linear change in A_1c_ at baseline and 3 and 6 months with group differences and prestudy A_1c_ level as a covariate.^a^

Factor		n	Usual care group	Treatment group	Group difference	*t*-ratio_159_^b^	*P*
**Mean A**_**1c**_ **level, % (mmol/mol)**							
	Baseline	90	8.16 (66)	8.46 (69)	0.30	1.57	.12
	3 months	83	7.68 (60)	7.81 (62)	0.11	0.63	.53
	6 months	80	7.46 (58)	7.35 (57)	–0.11	–0.59	.55
Prestudy A_1c_ effect^c^			0.52	—	—	5.23	<.001
**Instantaneous linear change in A**_**1c**_^d^							
	Baseline	90	–0.62	–0.80	–0.18	–0.94	.35
	3 months	83	–0.35	–0.56	–0.21	–1.87	.06
	6 months	80	–0.07	–0.31	–0.23	–2.87	.005
Estimated change from baseline to 6 months			–0.70	–1.11	–0.41		
Acceleration rate^e^			0.14	0.12	–0.01	–0.26	.80

^a^ Tabled values are maximum-likelihood estimates.

^b^ *t*-ratios are ratios of the estimates to their respective standard errors.

^c^ The prestudy A_1c_ effect, a regression coefficient, is the change in A_1c_ when measured during the study for a unit increase in prestudy A_1c_ level.

^d^ Instantaneous linear change in A_1c_ reflects the point change in A_1c_ for a 90-day increment.

^e^ Acceleration rate is the rate of acceleration of the quadratic growth model.

One usual care participant and 27 participants in the treatment group self-reported medication change, including 4 starting insulin. A change in medication was related to lower A_1c_ at 3 months (*t*_157_=–3.42, *P*<.001 and 6 months (*t*_157_=–3.37, *P*<.001) controlling for treatment effect and prestudy A_1c_. The effect of a medication change was not significant on the change rate in A_1c_ at 3 or 6 months (see Table 3). Three usual care group participants engaged with CDEs via telephone during the study. Although only 1 participant changed medication, they all decreased their A_1c_ levels.

The paired glucose testing goal was 84 pairs over 12 weeks with actual values ranging from zero to 73 pairs, mean 10.2 (SD 14.4) pairs, and median 21 (IQR 15) pairs. The effect of the number of paired glucose tests was not statistically significant on either the level (*t*_156_=–0.99, *P*=.33) or change rate in A_1c_ at 3 months (*t*_156_=–1.82, *P*=.07) and the level (*t*_156_=–1.86, *P*=.06) or change rate in A_1c_ at 6 months (*t*_156_=–1.82, *P*=.07) (see Table 3).

**Table 3:** Estimated effects of change in medication and number of paired glucose tests on A_1c_ level and instantaneous linear change in A_1c_ at baseline and at 3 and 6 months.

Predictor		Estimated effect on A_1c_ level^a^			Estimated effect on the instantaneous linear change rate in A_1c_		
		MLE^b^	*t*-ratio (*df*)^c^	*P*	MLE^b^	*t*-ratio (*df*)^c^	*P*
**Medication change**							
	3 months	–1.05	–3.42 (157)	<.001	0.35	1.81 (157)	.07
	6 months	–0.71	–3.37 (157)	<.001	0.35	1.81 (157)	.07
**Number of paired tests**							
	3 months	–0.007	–0.99 (156)	.33	–0.008	–1.82 (156)	.07
	6 months	–0.015	–1.86 (156)	.06	–0.008	–1.82 (156)	.07

^a^ Models were estimated in a hierarchical fashion.

^b^ MLE: maximum-likelihood estimate.

^c^ *t*-ratios are ratios of the estimates to their respective standard errors.

### Secondary Outcomes

Table 4 shows secondary outcomes by group over 3 months. Both groups showed improvement on average for general diet, specific diet, carbohydrate spacing, and foot care self-management behaviors measured by the SDSCA. The treatment group showed greater improvement in the self-management behaviors of carbohydrate spacing, monitoring glucose, and foot care. Neither group showed improvement in DKT, DES, or SDSCA subscales of exercise, smoking, and medication.

**Table 4:** Patient-reported secondary outcomes by group.

Secondary outcome^a^			Usual care		Treatment		*t*-ratio (*df*)	*P*
			n	Mean (95% CI)	n	Mean (95% CI)		
**DES**								
	Baseline		42	3.5 (3.3, 3.8)	45	3.8 (3.2, 4.4)	1.32 (85)	.19
	3 months		41	3.8 (3.2, 3.3)	40	4.1 (2.8, 5.3)^b^	0.39 (78)	.70
**DKT**								
	Baseline		42	12.0 (11.3, 12.6)	45	12.4 (10.9, 13.9)	0.98 (85)	.33
	3 months		41	11.4 (10.1, 12.6)	40	12.1 (9.1, 14.0)^b^	0.61 (78)	.55
**SDSCA**								
	**General diet**							
		Baseline	42	3.7 (3.2, 4.3)	45	3.7 (2.4, 5.0)	–0.07 (85)	.95
		3 months	41	4.9 (3.7, 6.1)	40	4.7 (2.0, 7.0)^b^	–0.20 (78)	.84
	**Specific diet**							
		Baseline	42	2.9 (2.4, 3.4)	45	3.5 (2.3, 4.7)	1.69 (85)	.09
		3 months	41	4.2 (3.0, 5.3)	40	4.6 (1.7, 7.0)^b^	–0.41 (78)	.68
	**Carbohydrate spacing**							
		Baseline	42	3.0 (2.3, 3.8)	45	2.7 (1.0, 4.6)	–0.46 (85)	.65
		3 months	41	3.9 (2.4, 5.4)	40	4.7 (3.4, 7.0)^b^	2.08 (78)	.04
	**Exercise**							
		Baseline	42	2.4 (1.7, 3.1)	45	2.7 (1.1, 4.3)	0.56 (85)	.58
		3 months	41	2.6 (1.3, 3.9)	40	3.7 (0.60, 6.8)	1.82 (78)	.07
	**Medication**							
		Baseline	42	6.5 (6.0, 7.0)	45	6.2 (4.9, 7.0)^b^	–0.93 (85)	.35
		3 months	41	6.4 (5.4, 7.3)	40	6.3 (5.2, 7.0)^b^	0.95 (78)	.34
	**Monitoring glucose**							
		Baseline	42	3.6 (2.8, 4.4)	45	3.0 (1.1, 4.8)	–1.17 (85)	.25
		3 months	41	3.7 (2.1, 5.3)	40	5.1 (2.6, 7.0)^b^	3.31 (78)	.001
	**Foot care**							
		Baseline	42	2.5 (1.8, 3.2)	45	2.5 (0.9, 4.2)	0.04 (85)	.97
		3 months	41	3.8 (2.5, 5.2)	40	5.0 (1.7, 7.0)^b^	2.42 (78)	.02

^a^ DES: Diabetes Empowerment Scale; DKT: Diabetes Knowledge Test; SDSCA: Summary of Diabetes Self-Care Activities.

^b^ Confidence intervals assume symmetry about the mean, reported with the maximum scale score as the upper bound contained within the interval estimate; DES max score=5.0; DKT max score=14.0; SDSCA max score=7.0.

## Discussion

### Principal Findings

To our knowledge, this is the first telehealth remote monitoring study for type 2 diabetes, within a diabetes management program, that incorporated all essential elements of structured monitoring, including (1) identifying frequency of glucose testing, (2) participant use and response to data, (3) health care provider data interpretation, and (4) therapy modifications [12,13] with paired glucose testing [22]. Structured monitoring created actionable patient-generated data in the context of a complete feedback loop facilitating change in both self-management behavior and treatment. In this study, both groups had improved A_1c_ levels at 3 months without a significant difference between groups in the rate of change (*P*=.06). However, at 6 months, the treatment group continued to have a statistically significant decrease in A_1c_ levels (demonstrating sustained benefit from the intervention), whereas the usual care group participants were no longer improving (*P*=.005). Both groups had lower A_1c_ levels with an estimated average decrease of 0.70 percentage points in the usual care group and 1.11 percentage points in the treatment group, with a significant group difference of 0.41 percentage points at 6 months. Although baseline A_1c_ levels were higher in the treatment group, it was not significantly different from the usual care group. These outcomes are similar to a recent systematic review and meta-analysis that demonstrated a statistically significant and clinically relevant mean difference in A_1c_ of -0.44 percentage points (-4.8 mmol/mol) between treatment and usual care when telehealth was added to usual care in diabetes management [30]. Previously, a difference of -0.50 percentage points in A_1c_ levels between treatment and usual care groups has been reported as clinically meaningful in the literature [14]. Implementation of all complete feedback loop elements (telehealth remote monitoring, structured SMBG, nurse care coordination, and treatment change) is necessary to improve outcomes and future clinical translational research needs to be conducted in the context of the complete feedback loop [20].

Reducing clinical inertia in management of type 2 diabetes was a goal of this intervention [3]. In this study, treatment participants had more self-reported medication changes compared to usual care participants and this was significantly associated with A_1c_ level at both 3 (*P*<.001) and 6 months (*P*<.001). The weekly asynchronous virtual visits provided analyzed glucose data to reinforce ADA goals and facilitate pattern management. The paired glucose testing analysis reduced clinical inertia for both patients and providers. Evaluating multiple weeks of SMBG data, often continuously above goal, compared to the usual practice of assessing a single A_1c_ result at 3-month intervals, encouraged medication change. This study is similar to others that incorporated nurse care coordination [25,31,32] to suggest medication changes to primary care providers, but different from those that used nurse practitioners [33,34] who were able to adjust medication independently. Although CDEs suggested medication changes, the primary care providers did not always follow the recommendations or ordered a different medication class. Allowing CDEs to adjust and order medications independently might improve outcomes. Although primary care providers had access to paired glucose testing data analysis, several chose to wait for A_1c_ results before initiating medication change. Targeted primary care provider education before the study may have improved outcomes and increased comfort level with adjusting medications using SMBG data [35].

In the telehealth literature, some studies report an association between the frequency of SMBG and the impact on A_1c_ change [33,36], whereas others report no impact [34]. The STeP study showed structured SMBG data resulted in more frequent and effective treatment changes by primary care providers [6]. The St Carlos [7], ROSSO [37], and PRISMA [17] studies also showed improvement in A_1c_ when structured SMBG data were used to adjust treatment. In this study, more sets of paired glucose testing were not associated with a faster rate of decline in A_1c_ levels (*P*=.06). However, the frequency of paired glucose testing varied. Identifying opportunities to encourage consistent paired glucose testing may improve outcomes. Research to examine the minimum number of paired glucose tests required to improve outcomes is important. The study was not powered to conduct subanalyses of individuals with consistent weekly paired glucose testing. Newer glucose monitoring technologies, including continuous glucose sensors that collect and store glucose data with minimal fingerstick requirements, may improve primary care provider and patient access to glucose data and reduce clinical inertia. Finally, long-term use of paired glucose testing over 12 months needs to be evaluated. Although A_1c_ data were collected at 6 months, the active intervention ended at 3 months. It is possible that continuing the telehealth remote monitoring would further improve outcomes. A comparative effectiveness study varying the frequency of paired glucose testing and type of remote monitoring feedback is necessary to identify best practices to lower A_1c_ levels [38].

This telehealth study incorporated asynchronous virtual encounters via the EHR to provide feedback on the weekly analysis of paired glucose testing data. This study was similar to others that focused on the use of EHRs for feedback [33,39,40], allowing participants to engage in self-management at a convenient time. This study was also similar to others that analyzed data using treatment algorithms [32], but offered a unique approach by using paired glucose testing data to educate the participant on glucose pattern management and empower participants in self-management.

The treatment group improved in 3 self-management behaviors. Daily education on AADE7 self-care behaviors required the participant to respond through the Care Innovations Guide [5]. The treatment group showed greater improvement in SDSCA self-management subscores of foot care, carbohydrate spacing, and monitoring glucose, all content areas presented in the Care Innovations Guide. The asynchronous virtual visits concentrated on the impact of paired glucose testing and helped participants evaluate carbohydrate quantity in food choices while identifying effect on glucose. This telehealth remote monitoring study is unique in that the participants were taught to analyze paired glucose testing data in the same manner as CDEs, looking for trends and patterns, and adjusting behavior accordingly. Data from a recent systematic review showed that only 31.1% of mobile apps create an opportunity for people with diabetes to share glucose data with providers and even fewer (17.8%) offer an opportunity to analyze data and graphically display results to help the individual learn from their data, whereas only 8.8% of applications supported patients in developing personalized action plans [38]. This study improved on limitations identified in this systematic review by sharing glucose data with the health care team, providing both automated and tailored feedback along with problem solving and goal setting support. Although foot care was not directly an intervention component, foot care was incorporated into the 84 virtual health sessions participants completed through the Care Innovations Guide. Participants improved scores in foot care due to the daily educational content provided through the Care Innovations Guide, which indicates virtual education is a successful option to improve self-management behaviors.

### Limitations

Due to challenges with recruitment and saturation of the participant pool, the sample size was small. Results may be different for larger studies powered to detect a smaller change in A_1c_ levels. The study took place over 6 months, thus long-term outcomes and sustainability over time is unknown. Although there are baseline data from the SDSCA subscale on glucose monitoring, we do not know if the usual care group engaged in paired glucose testing or another profile. However, most participants self-reported not checking glucose on a regular schedule and randomization would account for this issue. A Hawthorne effect is always possible when enrolling individuals motivated to change their self-management behaviors. A delayed entry or a crossover study would address the problem of usual care participants knowing group assignment. The population for the study was an existing diabetes management program, a higher level of usual care than described in most telehealth remote monitoring studies, so between-group differences may be smaller than if compared to typical usual care. Data were not collected on specific dietary or physical activity changes participants made. An online food diary and accelerometer to automatically capture physical activity would create a richer dataset to analyze. Although a treatment fidelity plan was in place, the same CDEs were responsible for treatment and usual care groups, possibly contaminating the usual care group. This intervention did not use mobile technology and was unable to provide real-time feedback, which may have limited outcomes [32].

This study enrolled insured, English-speaking participants in a diabetes management program in a health system. There may be membership bias because the study was conducted in English, yet people affected by diabetes represent multiple ethnic groups, many of whom are underinsured or uninsured. A majority of the participants were white, highly educated, with a strong history of computer use. The results of this study can only be generalized to a similar population. This study should be repeated in populations of lower socioeconomic status without access to sophisticated diabetes management programs. Varying the length of telehealth remote monitoring interventions, including duration and intensity, may help define specific requirements to improve outcomes [41]. There are significant costs associated with SMBG that impact patients and payers and a cost-effectiveness analysis would have provided important information. This intervention required 2 test strips per day whereas Medicare allows for 1 strip per day. Nurse care coordination is expensive and other technology-enabled models of care that facilitate the complete feedback loop and increase patient engagement at a lower cost are needed. Incorporating social media for patient support may reinforce problem solving and behavior change and be less costly. Weitzman and colleagues [42] identified that 31.7% of study participants posted their A_1c_ values on their profile page during a study in the diabetes online community Tudiabetes.org indicating an interest in participating in online peer support for glucose management.

### Conclusions

This eHealth clinical trial implemented essential elements of structured monitoring in tandem with telehealth remote monitoring and asynchronous virtual visits through a health system EHR. The complete feedback loop was utilized to educate participants, obtain and analyze actionable SMBG data, provide feedback, and collaborate as a team in the decision-making process. Participants used paired glucose testing data to change behavior, self-reporting an increase in both carbohydrate spacing and glucose monitoring. CDEs, experienced in pattern management and medication adjustment, suggested treatment changes in a reasonable time frame (typically 4 weeks), breaking a link in the cycle of clinical inertia, showing a change in medication was associated with a lower A_1c_ level [3]. At present, this level of nurse care coordination has limited reimbursement. Further research is needed to support eHealth models of care that incorporate remote nurse care coordination by CDEs [43]. Implementing a complete feedback loop in the primary care setting, supported by telehealth remote monitoring and paired glucose testing, improves A_1c_ and self-management behaviors in adults with type 2 diabetes.

## Appendix

Multimedia Appendix 1 CONSORT-EHEALTH checklist V1.6.2 [44].

Multimedia Appendix 2 Greenwood clinical inertia RCT PowerPoint presentation.

## Abbreviations

AADE: American Association of Diabetes Educators
ADA: American Diabetes Association
CDE: certified diabetes educators
DES: Diabetes Empowerment Scale short form
DKT: Diabetes Knowledge Test
DSMB: Data and Safety Monitoring Board
EHR: electronic health record
SDSCA: Summary of Diabetes Self-Care Activities
SMBG: self-monitoring of blood glucose
