# Supplementary material for: Overcoming Clinical Inertia: A Randomized Clinical Trial of a Telehealth Remote Monitoring Intervention Using Paired Glucose Testing in Adults With Type 2 Diabetes
Source: J Med Internet Res. 2015 Jul 21;17(7):e178. doi: 10.2196/jmir.4112 (PMC4527012; doi:10.2196/jmir.4112)
Supplement: Multimedia Appendix 1 [file jmir_v17i7e178_app1.pdf]

CONSORT-EHEALTH Checklist V1.6.2 Report

(based on CONSORT-EHEALTH V1.6), available at [http://tinyurl.com/consort-ehealth-v1-6].

4112

Date completed

12/22/2014 0:49:57

by

Deborah Greenwood

Overcoming Clinical Inertia: A Randomized Clinical Trial of a Telehealth Remote Monitoring Intervention using Paired Glucose Testing in Adults with Type 2 Diabetes

TITLE

1a-i) Identify the mode of delivery in the title

Telehealth remote monitoring is a combination of delivery modes and was not mobile

1a-ii) Non-web-based components or important co-interventions in title

remote monitoring

1a-iii) Primary condition or target group in the title

Adults with Type 2 Diabetes

ABSTRACT

1b-i) Key features/functionalities/components of the intervention and comparator in the METHODS section of the ABSTRACT

"The telehealth RM tablet computer transmitted glucose data and facilitated a CFL to educate participants, analyze actionable glucose data, and provide feedback. Data from paired glucose testing were analyzed asynchronously using computer-assisted pattern analysis and were shared with patients via the EHR weekly. CDEs called participants monthly to discuss paired glucose testing trends and treatment changes. "

1b-ii) Level of human involvement in the METHODS section of the ABSTRACT

"paired glucose testing were analyzed asynchronously using computer-assisted pattern analysis"

1b-iii) Open vs. closed, web-based (self-assessment) vs. face-to-face assessments in the METHODS section of the ABSTRACT

"The telehealth RM tablet computer transmitted glucose data

1b-iv) RESULTS section in abstract must contain use data

Not applicable we were not evaluating

1b-v) CONCLUSIONS/DISCUSSION in abstract for negative trials

outcomes were not negative

INTRODUCTION

2a-i) Problem and the type of system/solution

"Practice guidelines promote the use of SMBG for informing healthcare providers about glycemic control and providing patient feedback to increase knowledge, self-efficacy, and behavior change"

"A complete feedback loop (CFL)—data collection and interpretation combined with feedback to the patient to modify treatment plans—has been associated with improved outcomes [19]. While the CFL has been identified as an essential component of both SMBG [20] and remote monitoring (RM), there remains limited and inconsistent incorporation of SMBG feedback in diabetes management "

2a-ii) Scientific background, rationale: What is known about the (type of) system

"Telehealth RM may improve clinical outcomes, care coordination, engagement and satisfaction"

METHODS

3a) CONSORT: Description of trial design (such as parallel, factorial) including allocation ratio

"The purpose of this study was to test the effectiveness of a telehealth RM intervention with paired glucose testing for adults with noninsulin treated type 2 diabetes. The hypothesis was that a telehealth RM intervention with paired glucose testing would result in a greater change in A1C, improved self-management, self-efficacy, and diabetes knowledge compared to usual care over six months. "

3b) CONSORT: Important changes to methods after trial commencement (such as eligibility criteria), with reasons

No changes

3b-i) Bug fixes, Downtimes, Content Changes

N/A no issues to report

4a) CONSORT: Eligibility criteria for participants

"Inclusion criteria:
• Type 2 diabetes diagnosis treated with oral anithyperglycemic medications or lifestyle alone,
• Participant in the diabetes management program for previous 12 months,
• 30-70 years of age,
• A1C between 7.5% and 10.9% in previous six months,
• Internet or 3G connection with e-mail access,
• Land line or cellular phone,
• English speaking.
• PCP in health system.
Exclusion criteria identified by medical chart review including:
• Insulin prescription,
• Unable to independently self-manage (diagnosis of dementia, severe depression, schizophrenia, or cognitive impairment for previous 12 months),
• Diagnoses of stroke, heart failure, end-stage renal disease, or legally blind.
"

4a-i) Computer / Internet literacy

"Internet or 3G connection with e-mail access, "

4a-ii) Open vs. closed, web-based vs. face-to-face assessments:

"Eligible participants were contacted through e-mail, postal mail, and telephone. (See Figure 1). Consent forms were e-mailed and mailed to participants with informed consent conducted over the telephone and consent forms returned by mail."

4a-iii) Information giving during recruitment

Eligible participants were contacted through e-mail, postal mail, and telephone. (See Figure 1). Consent forms were e-mailed and mailed to participants with informed consent conducted over the telephone and consent forms returned by mail.

4b) CONSORT: Settings and locations where the data were collected

The study was conducted between January and October in 2013 in a large healthcare system in California with an established diabetes management program incorporating telephonic nurse care coordination for diabetes population management.

4b-i) Report if outcomes were (self-)assessed through online questionnaires

"After participants completed online baseline self-assessment questionnaires they were notified of their study group assignment by e-mail."

4b-ii) Report how institutional affiliations are displayed

This information was included in the consent form and not documented in paper

5) CONSORT: Describe the interventions for each group with sufficient details to allow replication, including how and when they were actually administered

5-i) Mention names, credential, affiliations of the developers, sponsors, and owners

"The 84 sequential daily health sessions, designed by the research team, were delivered through the Guide as a text document in the style of a PowerPoint slide or via short video clips."

5-ii) Describe the history/development process

Content was based on written material that already existed.

5-iii) Revisions and updating

there are no revisions to report

5-iv) Quality assurance methods

Training provided for quality control

5-v) Ensure replicability by publishing the source code, and/or providing screenshots/screen-capture video, and/or providing flowcharts of the algorithms used

"Figure 2. Weekly paired glucose testing data analysis, by software designed for the study, for feedback to participants through asynchronous secure messaging via the EHR"

Clinical decision making by health care providers

5-vi) Digital preservation

there is no URL to archive.

5-vii) Access

"Participants in both groups were offered a $10 gift card after completing online questionnaires. "
"A1C tests were ordered every three months, as is standard of care when A1C is elevated, and then billed to insurance. All A1Cs were collected at health system labs using similar equipment following standardized procedures. All questionnaires were completed online, using the REDCap (Research Electronic Data Capture) database. "
"Participants received education on how to use the Care Innovations Guide™ (Guide), a U.S. Food and Drug Administration (FDA) approved telehealth RM system that combines an in-home tablet computer connected by Internet or 3G network to the Care Innovations Health Suite ™ (Health Suite) online portal (Care Innovations, an Intel-GE company, Roseville, CA, USA). The Guide is connected to the glucometer via USB cables and has a touch-screen for participants to answer daily health session questions. Data are downloaded to the Health Suite for CDEs to access via the Internet. Participants received a OneTouch® Ultra® 2 glucometer (approved by the FDA) test supplies, and USB cables to keep (Johnson and Johnson-Lifescan Inc., Milpitas, CA, USA) at no charge. Participants returned the Guide when the intervention concluded. "

5-viii) Mode of delivery, features/functionalities/components of the intervention and comparator, and the theoretical framework

This information is located in the following sections
"Treatment Group
Structured glucose monitoring. "
and
"Data review and nurse care coordination."

5-ix) Describe use parameters

This information is located in the following sections
"Treatment Group
Structured glucose monitoring. "
and
"Data review and nurse care coordination."

5-x) Clarify the level of human involvement

This information is located in the following sections
"Treatment Group
Structured glucose monitoring. "
and
"Data review and nurse care coordination."

5-xi) Report any prompts/reminders used

This information is located in the following sections
"Treatment Group
Structured glucose monitoring. "
and
"Data review and nurse care coordination."

5-xii) Describe any co-interventions (incl. training/support)

"Prior to the intervention, CDEs attended in-person training sessions on intervention procedures, paired testing and the goal of implementing a CFL. Participants attended a one hour in-person training that included: (1) education regarding use of glucose meter; (2) implementation of the CFL; (3) use of paired glucose testing (frequency and intensity of monitoring); (4) ADA goals for pre- and postmeal; (5) how to use SMBG data to modify behavior or treatment; (6) expected feedback from CDEs and communication by secure message or phone; and (7) the use of shared decision making to implement treatment plan [22]. Participants created a "personal experiment" and agreed to check glucose before and two hours after the same meal or physical activity for one week, and created a behavior change action plan to evaluate changes in SMBG data. "

6a) CONSORT: Completely defined pre-specified primary and secondary outcome measures, including how and when they were assessed

The following information is located in the section
"Measures
Primary Outcome"
and "Secondary Outcomes"

6a-i) Online questionnaires: describe if they were validated for online use and apply CHERRIES items to describe how the questionnaires were designed/deployed
"Diabetes Knowledge. Diabetes knowledge was measured using the Diabetes Knowledge Test (DKT) [26], a valid and reliable measure for estimating general understanding of diabetes, including healthy eating and glucose monitoring consisting of 23 multiple choice items. The first 14 items, appropriate for people not using insulin, were administered to study participants. Scores are measured as the number of correct answers divided by the possible total of 14.
Self-management. Self-management was measured by the Summary of Diabetes Self-Care Activities (SDSCA) [27], a 12-item self-report questionnaire with subscales: general diet, specific diet, carbohydrate spacing, exercise, monitoring blood glucose, and foot care. Participants were asked "In how many of the past seven days (0-7) did you check your blood glucose?" etc. Higher scores indicate better self-care behavior.
Self-efficacy. Self-efficacy was measured by the Diabetes Empowerment Scale short form (DES-SF), an eight-item measure of psychosocial self-efficacy in people with diabetes [28]. Scores ranged from 1–5, with 5 indicating "strongly agree." Mean score of eight items is reported."

6a-ii) Describe whether and how "use" (including intensity of use/dosage) was defined/measured/monitored
"The 84 sequential daily health sessions"
"The paired glucose testing goal was 84 pairs over 12 weeks with actual values ranging from zero to 73 pairs with a mean of 10.2 (SD 14.4) pairs and median of 21 pairs. "

6a-iii) Describe whether, how, and when qualitative feedback from participants was obtained
this was a quantitative study so there are no qualitative results to report

6b) CONSORT: Any changes to trial outcomes after the trial commenced, with reasons
No changes to trial outcomes

7a) CONSORT: How sample size was determined

7a-i) Describe whether and how expected attrition was taken into account when calculating the sample size
"Sample size was determined based on the main outcome, mean change in A1C between intervention and control over six months. The comparison of usual care (n = 39) to intervention participants (n = 39) has 80% power to detect a 0.9% difference in A1C between intervention and control after 6 months (α = 0.05, 2-tailed). A 15% additional margin for participant drop-out resulted in a sample size of n=45 per group."

7b) CONSORT: When applicable, explanation of any interim analyses and stopping guidelines
There were no interim analysis

8a) CONSORT: Method used to generate the random allocation sequence
"A permuted block, with blocks of four and six, and a computer-generated random number table were utilized for randomization. Once participants signed the consent form, they were assigned sequential research study identification (ID) numbers by the research coordinator and then the investigator matched the ID numbers to the random number table to assign study group. After participants completed online baseline self-assessment questionnaires they were notified of their study group assignment by e-mail. "

8b) CONSORT: Type of randomisation; details of any restriction (such as blocking and block size)
"A permuted block, with blocks of four and six, and a computer-generated random number table were utilized for randomization. Once participants signed the consent form, they were assigned sequential research study identification (ID) numbers by the research coordinator and then the investigator matched the ID numbers to the random number table to assign study group. After participants completed online baseline self-assessment questionnaires they were notified of their study group assignment by e-mail. "

9) CONSORT: Mechanism used to implement the random allocation sequence (such as sequentially numbered containers), describing any steps taken to conceal the sequence until interventions were assigned
"A permuted block, with blocks of four and six, and a computer-generated random number table were utilized for randomization. Once participants signed the consent form, they were assigned sequential research study identification (ID) numbers by the research coordinator and then the investigator matched the ID numbers to the random number table to assign study group. After participants completed online baseline self-assessment questionnaires they were notified of their study group assignment by e-mail. "

10) CONSORT: Who generated the random allocation sequence, who enrolled participants, and who assigned participants to interventions
"A permuted block, with blocks of four and six, and a computer-generated random number table were utilized for randomization. Once participants signed the consent form, they were assigned sequential research study identification (ID) numbers by the research coordinator and then the investigator matched the ID numbers to the random number table to assign study group. After participants completed online baseline self-assessment questionnaires they were notified of their study group assignment by e-mail. "

11a) CONSORT: Blinding - If done, who was blinded after assignment to interventions (for example, participants, care providers, those assessing outcomes) and how

11a-i) Specify who was blinded, and who wasn't
"Blinding of participants, providers, or research team was not possible. "

11a-ii) Discuss e.g., whether participants knew which intervention was the "intervention of interest" and which one was the "comparator"
"Blinding of participants, providers, or research team was not possible. "

11b) CONSORT: If relevant, description of the similarity of interventions
"Usual Care
Participants in usual care received diabetes education booklets and referral for formal diabetes education as needed. This group continued to receive nurse care coordination with reminders for A1C and health maintenance exams sent by postal mail. The CDEs evaluated SMBG data when reported by participants (no specific monitoring profile is required) and discussed behavior changes with participants by telephone and/or secure messaging and discussed possible medication changes with the PCP through the EHR staff messaging tool. Contact with usual care was documented in the study database.
"

12a) CONSORT: Statistical methods used to compare groups for primary and secondary outcomes
" Mixed-effects models were used to compare mean change over time in primary and secondary outcomes between groups. A1C was measured at baseline and then at approximately three and six months."
"SAS version 9.4 was used to obtain restricted maximum likelihood estimates using PROC MIXED. "

12a-i) Imputation techniques to deal with attrition / missing values
Mixed effects models allow for missing data and were most suited for this study.

12b) CONSORT: Methods for additional analyses, such as subgroup analyses and adjusted analyses
". An indicator of group membership was added to the best fitting growth model to test for differences between groups with regard to A1C at three and six months and the change in A1C, statistically adjusting for pre-study A1C. An indicator variable was added to the model that denoted whether a participant experienced a change in medication during the study and tested the effect of medication change on A1C at three and six months, and on the change in A1C. Finally, a model was fit to test the effect of the number of paired glucose tests on A1C at three and six months and change in A1C over time, controlling for change in medication and pre-study A1C. The effect of the number of paired glucose tests on A1C, accounting for effects due to changes in medication, was studied. Tests used a significance level of 0.05 or 95% CI that excludes zero. "

RESULTS

13a) CONSORT: For each group, the numbers of participants who were randomly assigned, received intended treatment, and were analysed for the primary outcome
Data are described in results section and numbers of participants analyzed are documented in the tables.

13b) CONSORT: For each group, losses and exclusions after randomisation, together with reasons

Consort flow chart

13b-i) Attrition diagram

Consort flow chart

14a) CONSORT: Dates defining the periods of recruitment and follow-up

"The study was conducted between January and October in 2013 in a large healthcare system in California with an established diabetes management program incorporating telephonic nurse care coordination for diabetes population management. "
paper states the participants were followed for 6 months.

14a-i) Indicate if critical "secular events" fell into the study period

There were no secular events

14b) CONSORT: Why the trial ended or was stopped (early)

The trial was not stopped early.
Recruitment was a challenge and ended with 90 enrolled.

15) CONSORT: A table showing baseline demographic and clinical characteristics for each group

Table 1

15-i) Report demographics associated with digital divide issues

Table 1

16a) CONSORT: For each group, number of participants (denominator) included in each analysis and whether the analysis was by original assigned groups

16-i) Report multiple "denominators" and provide definitions

primary and secondary outcomes were intent to treat.
Additional analyses were performed on the intervention group and reported after the primary outcomes.

16-ii) Primary analysis should be intent-to-treat

primary and secondary outcomes were intent to treat.
"All data were included in an intent-to-treat analysis. "

17a) CONSORT: For each primary and secondary outcome, results for each group, and the estimated effect size and its precision (such as 95% confidence interval)

yes, see tables 2-4

17a-i) Presentation of process outcomes such as metrics of use and intensity of use

"The paired glucose testing goal was 84 pairs over 12 weeks with actual values ranging from zero to 73 pairs with a mean of 10.2 (SD 14.4) pairs and median of 21 pairs. "

"Medication change was self-reported at 12 weeks by one participant from control and 27 from th treatment group, including four that started insulin. "

17b) CONSORT: For binary outcomes, presentation of both absolute and relative effect sizes is recommended

There are no binary outcomes

18) CONSORT: Results of any other analyses performed, including subgroup analyses and adjusted analyses, distinguishing pre-specified from exploratory

"The effect of the number of paired glucose tests was not statistically significant on either the level (estimated effect of -0.007, se = .007, t = -0.99, p = .33) or the change rate in A1C at three months (estimated effect of -0.008, se = .004, t = -1.82, p = .07) and the level (estimated effect of -0.015, se = .008, t = -1.86, p = .06) or the change rate in A1C at six months (estimated effect of -0.008, se = .004, t = -1.82, p = .07) (See Table 3). "

18-i) Subgroup analysis of comparing only users

"The effect of the number of paired glucose tests was not statistically significant on either the level (estimated effect of -0.007, se = .007, t = -0.99, p = .33) or the change rate in A1C at three months (estimated effect of -0.008, se = .004, t = -1.82, p = .07) and the level (estimated effect of -0.015, se = .008, t = -1.86, p = .06) or the change rate in A1C at six months (estimated effect of -0.008, se = .004, t = -1.82, p = .07) (See Table 3)."

19) CONSORT: All important harms or unintended effects in each group

There were none to report.
:There were no serious hyper or hypoglycemic events or hospitalizations. One participant visited the emergency-room unrelated to the study and DSMB determined there were no serious adverse events related to the study."

19-i) Include privacy breaches, technical problems

There were none to report

19-ii) Include qualitative feedback from participants or observations from staff/researchers

The outcomes of this study were quantitative so not reported.

DISCUSSION

20) CONSORT: Trial limitations, addressing sources of potential bias, imprecision, multiplicity of analyses

20-i) Typical limitations in ehealth trials

"Limitations. Due to challenges with recruitment and saturation of the participant pool, the sample size was small. Results may be different for larger studies powered to detect a smaller change in A1C. The study took place over six months, thus long-term outcomes and sustainability over time is unknown. Although there are data from the SDSCA subscale on glucose monitoring, we do not know if the control group engaged in paired glucose testing or another profile. However, most participants self-reported not checking glucose on a regular schedule and randomization would account for this issue. There may have been a Hawthorne effect enrolling individuals motivated to focus on changing their diabetes self-management behaviors. A delayed entry or a cross-over study would provide enhanced evidence by addressing the problem of control group participants knowing group assignment. The population for the study was an existing diabetes management program, a higher level of usual care than described in most telehealth RM studies, so between group differences may be smaller than if compared to typical usual care. Data were not collected on specific dietary or physical activity changes participants made. An online food-diary would create a richer data set to analyze. Although a treatment fidelity plan was in place, the same CDEs were responsible for intervention and control, possibly contaminating the control group. This intervention did not use mobile technology, and was unable to provide real-time feedback, which may have limited outcomes "

21) CONSORT: Generalisability (external validity, applicability) of the trial findings

21-i) Generalizability to other populations

" This study enrolled participants in a diabetes management program in a health system. The results of this study can only be generalized to a similar population. A majority of the participants were White, highly educated, with a strong history of computer use. "

21-ii) Discuss if there were elements in the RCT that would be different in a routine application setting

This was a real-world clinical environment and translational in nature

22) CONSORT: Interpretation consistent with results, balancing benefits and harms, and considering other relevant evidence

22-i) Restate study questions and summarize the answers suggested by the data, starting with primary outcomes and process outcomes (use)

"To our knowledge, this is the first telehealth RM study in type 2 diabetes, within a diabetes management program, that has incorporated all essential elements of structured monitoring, including: (a) identifying frequency of glucose testing, (b) participant use and response to data, (c) healthcare provider data interpretation, and (d) therapy modifications [12, 13] utilizing paired glucose testing[22]. Incorporating all essential elements provided actionable patient generated data in the context of a CFL enabling change in both behaviors and treatment. In this study, both groups improved A1C at three months without a significant difference between groups in the rate of change (P=0.06). However, at six months the intervention group continued to have a statistically significant decrease in A1C whereas the control group was no longer improving (P=0.005) demonstrating sustained benefit from the intervention. A clinically meaningful difference of 0.50 percentage points in A1C between intervention and control is often reported in the literature [14]. Both groups lowered A1C with an estimated average decrease of 0.70 percentage points in control and 1.11 percentage points in the intervention group, with a significant group difference of 0.41 percentage points at 6 months. Implementation of all CFL elements (telehealth RM, structured SMBG, nurse care coordination, and treatment change) is necessary to improve outcomes, and future clinical translational research needs to be conducted in the context of the CFL [20]. "

22-ii) Highlight unanswered new questions, suggest future research

" This study enrolled participants in a diabetes management program in a health system. The results of this study can only be generalized to a similar population. A majority of the participants were White, highly educated, with a strong history of computer use. This study should be repeated in populations of lower socioeconomic status without access to sophisticated diabetes management program. A comparative effectiveness study varying the frequency of paired glucose testing may provide information to identify the minimum number of paired glucose testing needed to lower A1C [40]. Varying the length of telehealth RM interventions, including duration and intensity, may help define specific requirements to improve outcomes [41]. Incorporating social media for patient support may reinforcement problem solving and behavior change. "

Other information

23) CONSORT: Registration number and name of trial registry

Clinicaltrials.gov identifier NCT01715649

24) CONSORT: Where the full trial protocol can be accessed, if available

There is no published protocol

25) CONSORT: Sources of funding and other support (such as supply of drugs), role of funders

"This research project has research support from the Investigator Initiated Studies program of LifeScan Corporation, Intel-GE Care Innovations, Sutter Institute for Medical Research, The Betty Irene Moore School of Nursing, The Jonas Center for Nursing Excellence, and the University of California Davis, National Center for Advancing Translational Sciences, National Institutes of Health, through grant number UL1 TR 000002. "

X26-i) Comment on ethics committee approval

". The study was approved by Sutter Health Central Institutional Review Committee and a Data and Safety Monitoring Board (DSMB) reviewed study procedures and adverse events."

x26-ii) Outline informed consent procedures

"Eligible participants were contacted through e-mail, postal mail, and telephone. (See Figure 1). Consent forms were e-mailed and mailed to participants with informed consent conducted over the telephone and consent forms returned by mail."

X26-iii) Safety and security procedures

This is addressed in the consent form that databases were HIPAA compliant.
"All questionnaires were completed online, using the REDCap (Research Electronic Data Capture) database. "

X27-i) State the relation of the study team towards the system being evaluated

"No other potential conflicts of interest were reported. D.A.G was the Principal Investigator for the study. The funders of this study did not play a role in the design and conduct of the study; collection, management, analysis, and interpretation of the data, or in the preparation of the manuscript. Intel-GE Care Innovations did not have veto power over or have say about changing any manuscript text other than the description of the hardware and software they provided. D.A.G. is the guarantor of this work, as such, had full access to all the data in the study and takes responsibility for the integrity of the data and the accuracy of the data analysis. "
