# Supplementary material for: Overcoming Clinical Inertia: A Randomized Clinical Trial of a Telehealth Remote Monitoring Intervention Using Paired Glucose Testing in Adults With Type 2 Diabetes
Source: J Med Internet Res. 2015 Jul 21;17(7):e178. doi: 10.2196/jmir.4112 (PMC4527012; doi:10.2196/jmir.4112)
Supplement: Multimedia Appendix 2 [file jmir_v17i7e178_app2.pptx]

## Slide 1
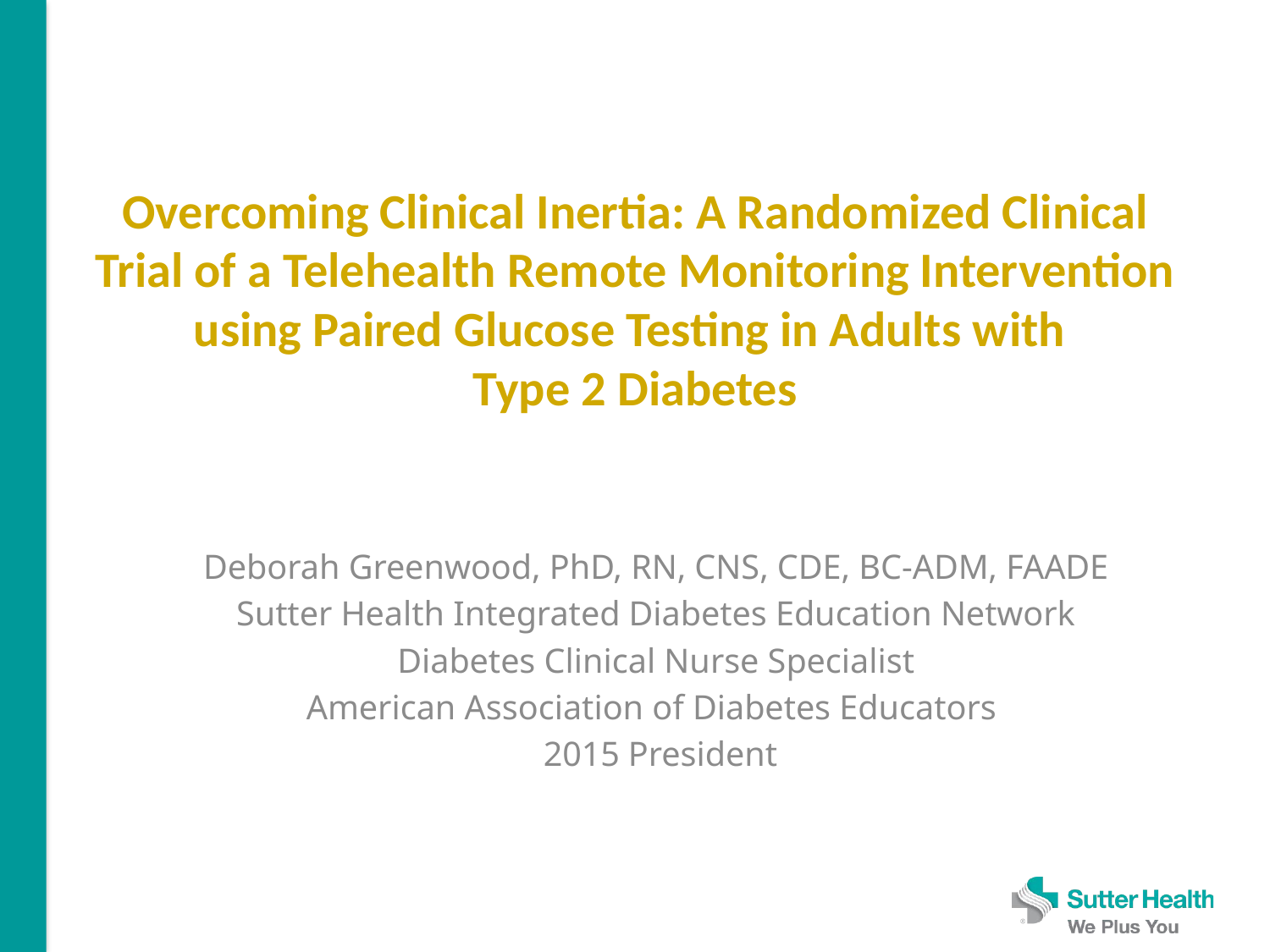

# Overcoming Clinical Inertia: A Randomized Clinical Trial of a Telehealth Remote Monitoring Intervention using Paired Glucose Testing in Adults with Type 2 Diabetes
Deborah Greenwood, PhD, RN, CNS, CDE, BC-ADM, FAADE
Sutter Health Integrated Diabetes Education Network
Diabetes Clinical Nurse Specialist
American Association of Diabetes Educators
 2015 President

## Slide 2
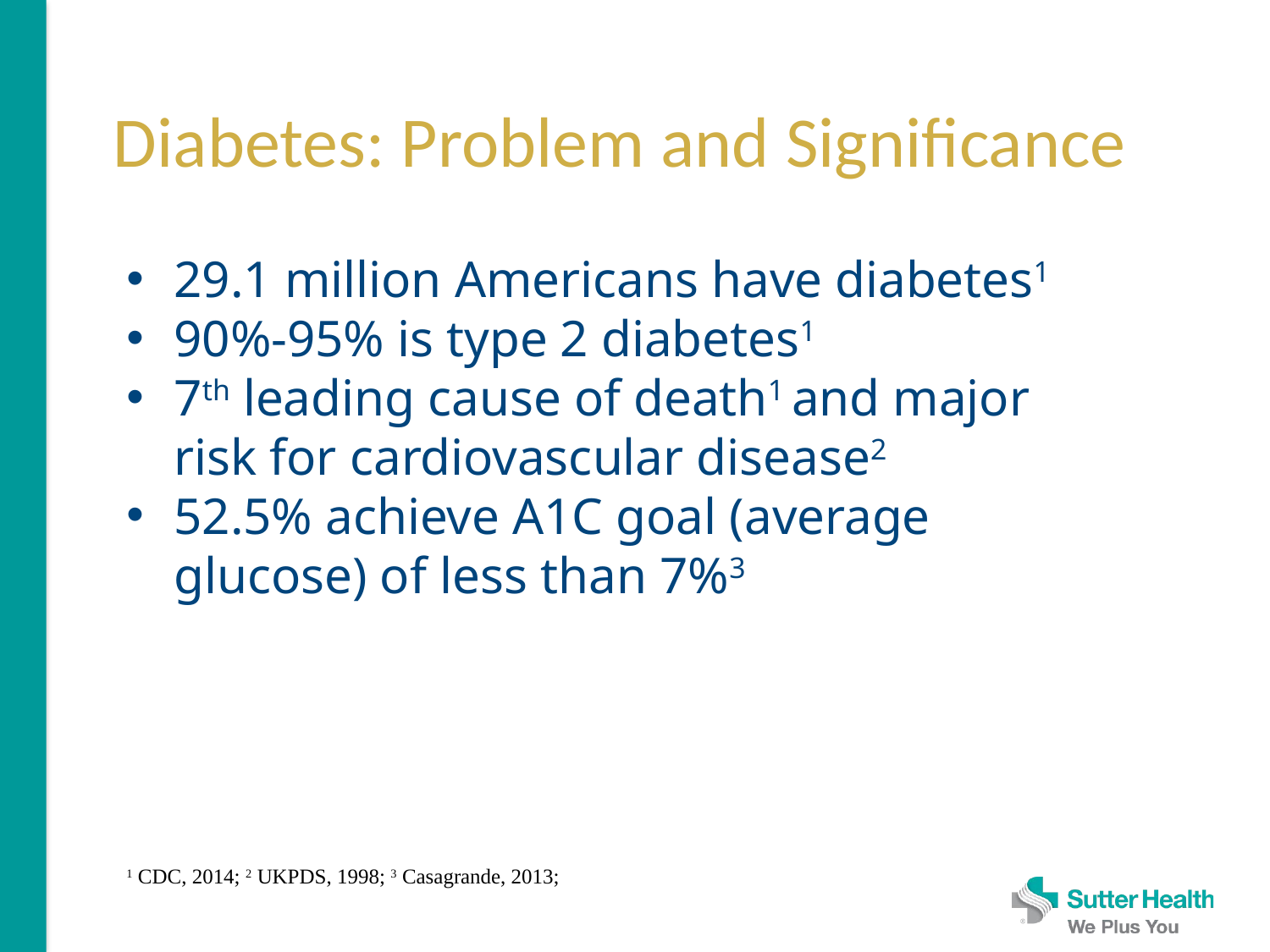

# Diabetes: Problem and Significance
29.1 million Americans have diabetes1
90%-95% is type 2 diabetes1
7th leading cause of death1 and major risk for cardiovascular disease2
52.5% achieve A1C goal (average glucose) of less than 7%3
1 CDC, 2014; 2 UKPDS, 1998; 3 Casagrande, 2013;

## Slide 3
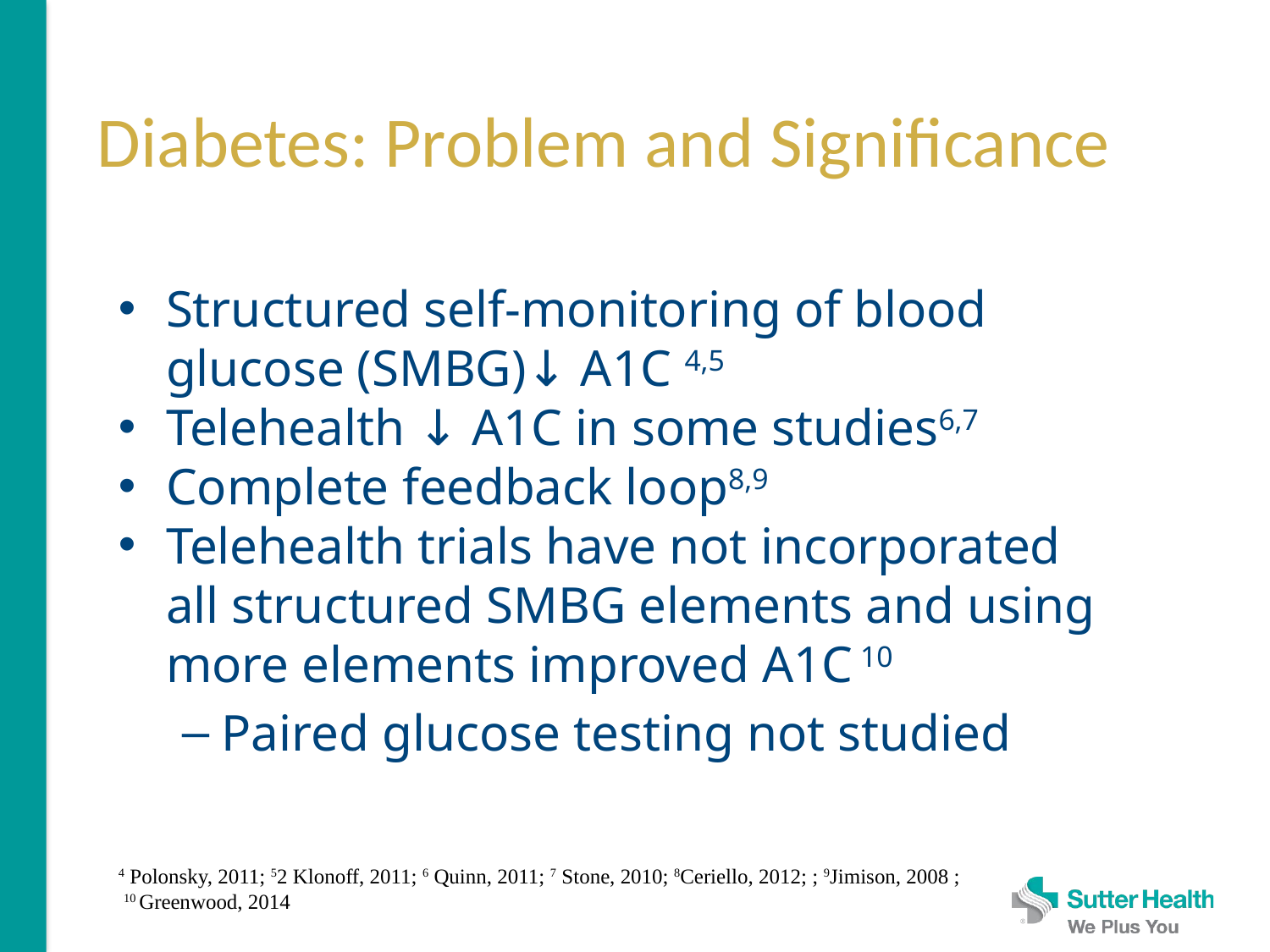

# Diabetes: Problem and Significance
Structured self-monitoring of blood glucose (SMBG)↓ A1C 4,5
Telehealth ↓ A1C in some studies6,7
Complete feedback loop8,9
Telehealth trials have not incorporated all structured SMBG elements and using more elements improved A1C 10
Paired glucose testing not studied
4 Polonsky, 2011; 52 Klonoff, 2011; 6 Quinn, 2011; 7 Stone, 2010; 8Ceriello, 2012; ; 9Jimison, 2008 ;
 10 Greenwood, 2014

## Slide 4
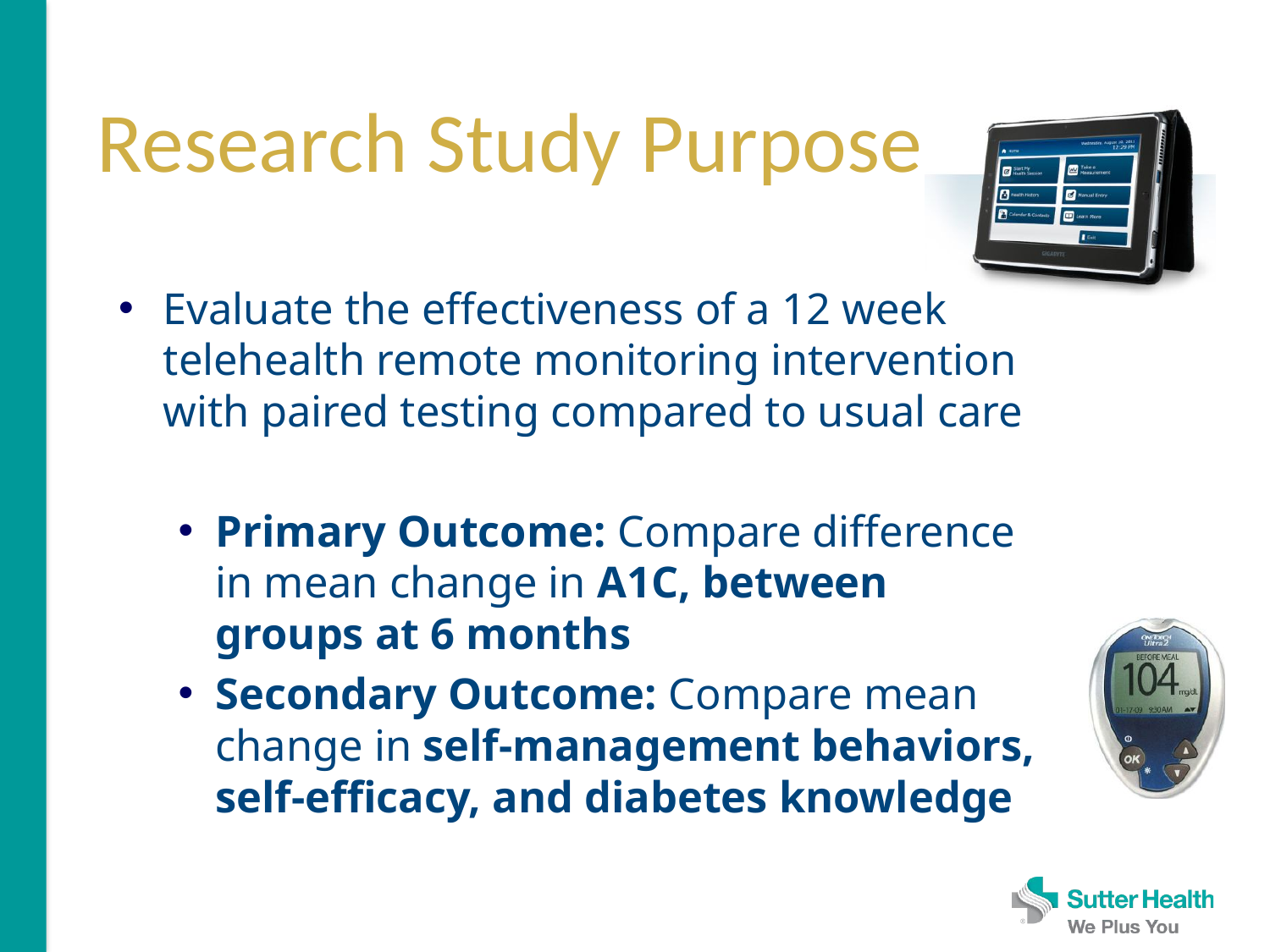

# Research Study Purpose
Evaluate the effectiveness of a 12 week telehealth remote monitoring intervention with paired testing compared to usual care
Primary Outcome: Compare difference in mean change in A1C, between groups at 6 months
Secondary Outcome: Compare mean change in self-management behaviors, self-efficacy, and diabetes knowledge

## Slide 5
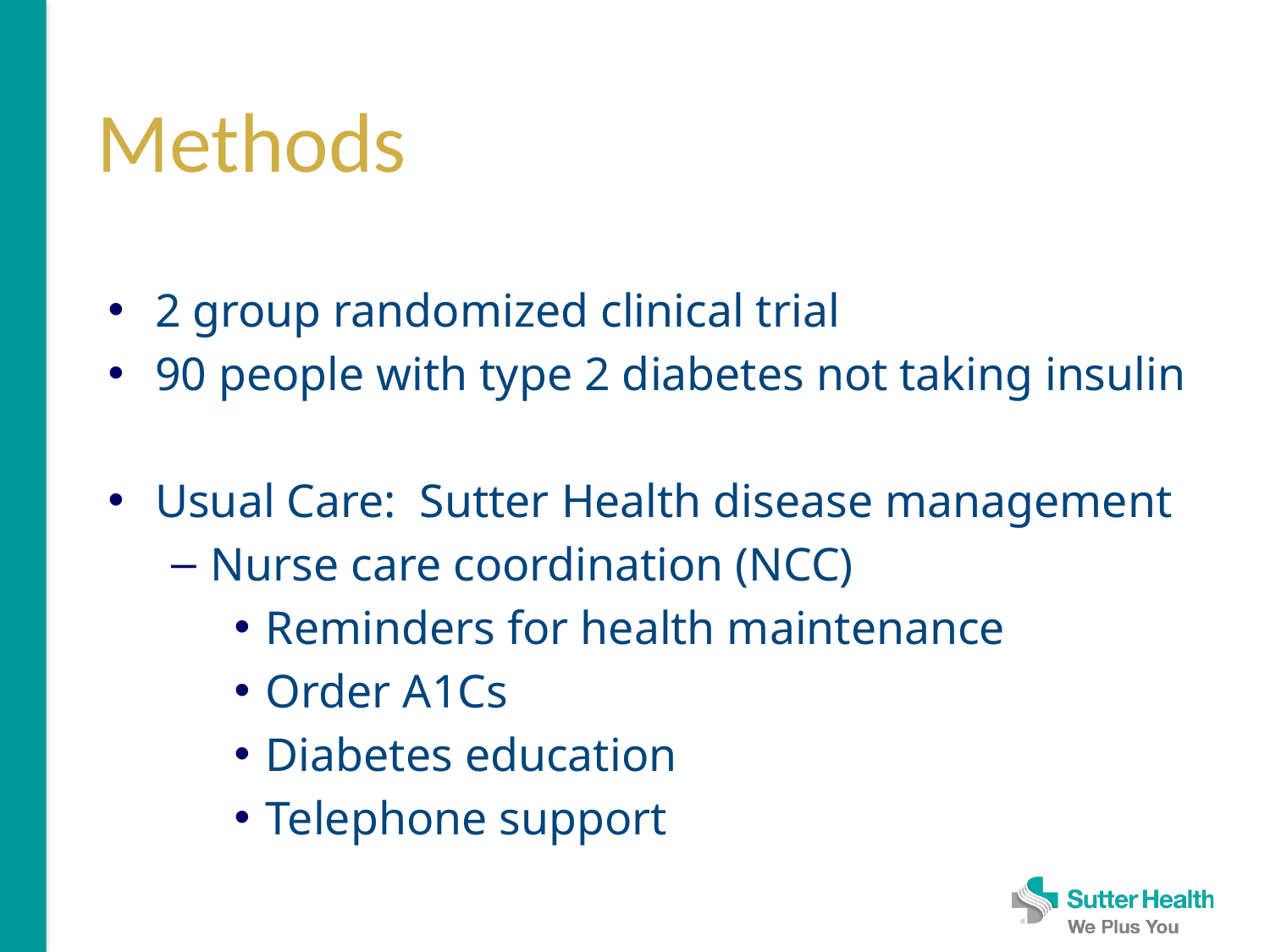

# Methods
2 group randomized clinical trial
90 people with type 2 diabetes not taking insulin
Usual Care: Sutter Health disease management
Nurse care coordination (NCC)
Reminders for health maintenance
Order A1Cs
Diabetes education
Telephone support

## Slide 6
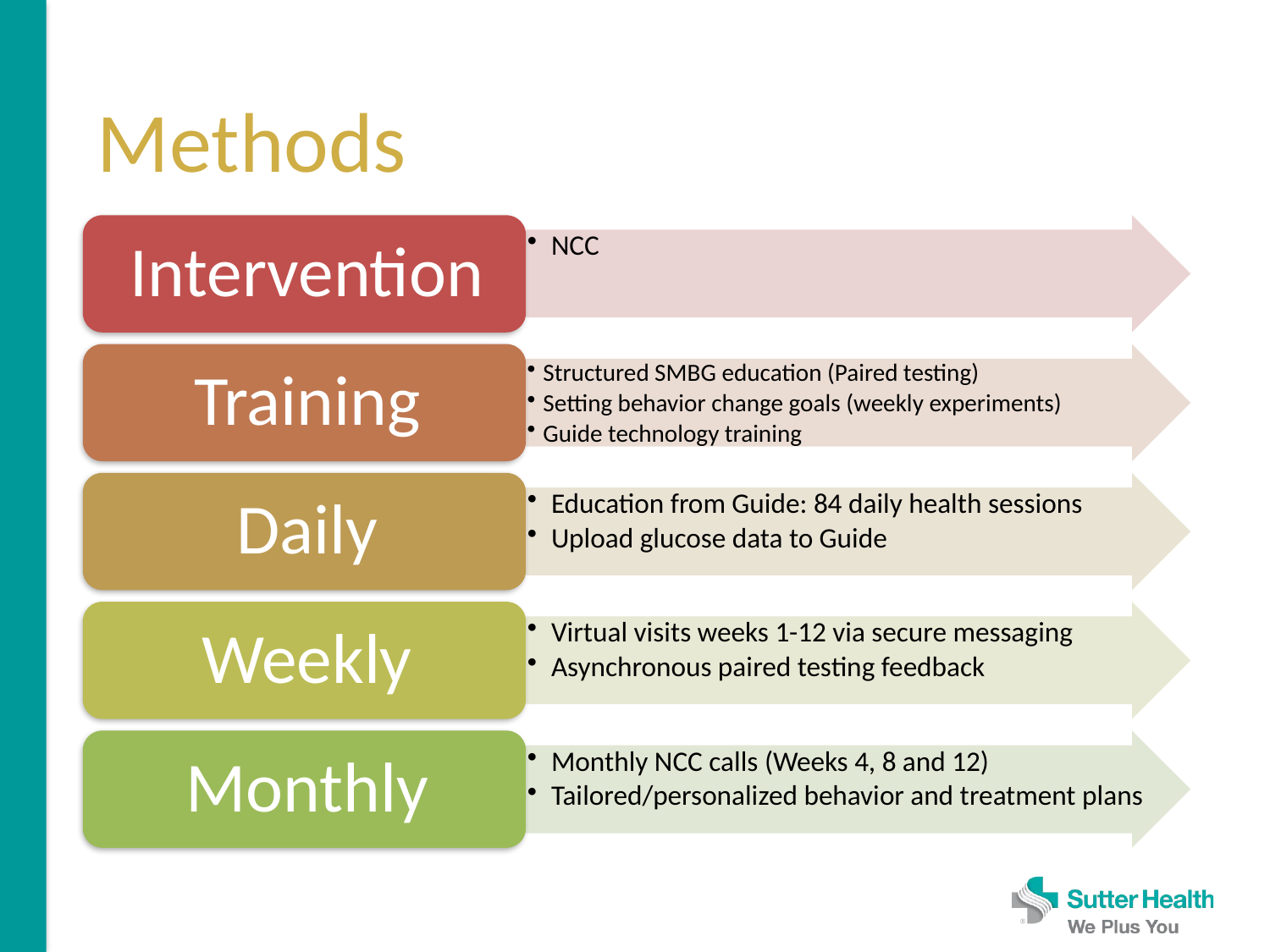

# Methods

## Slide 7
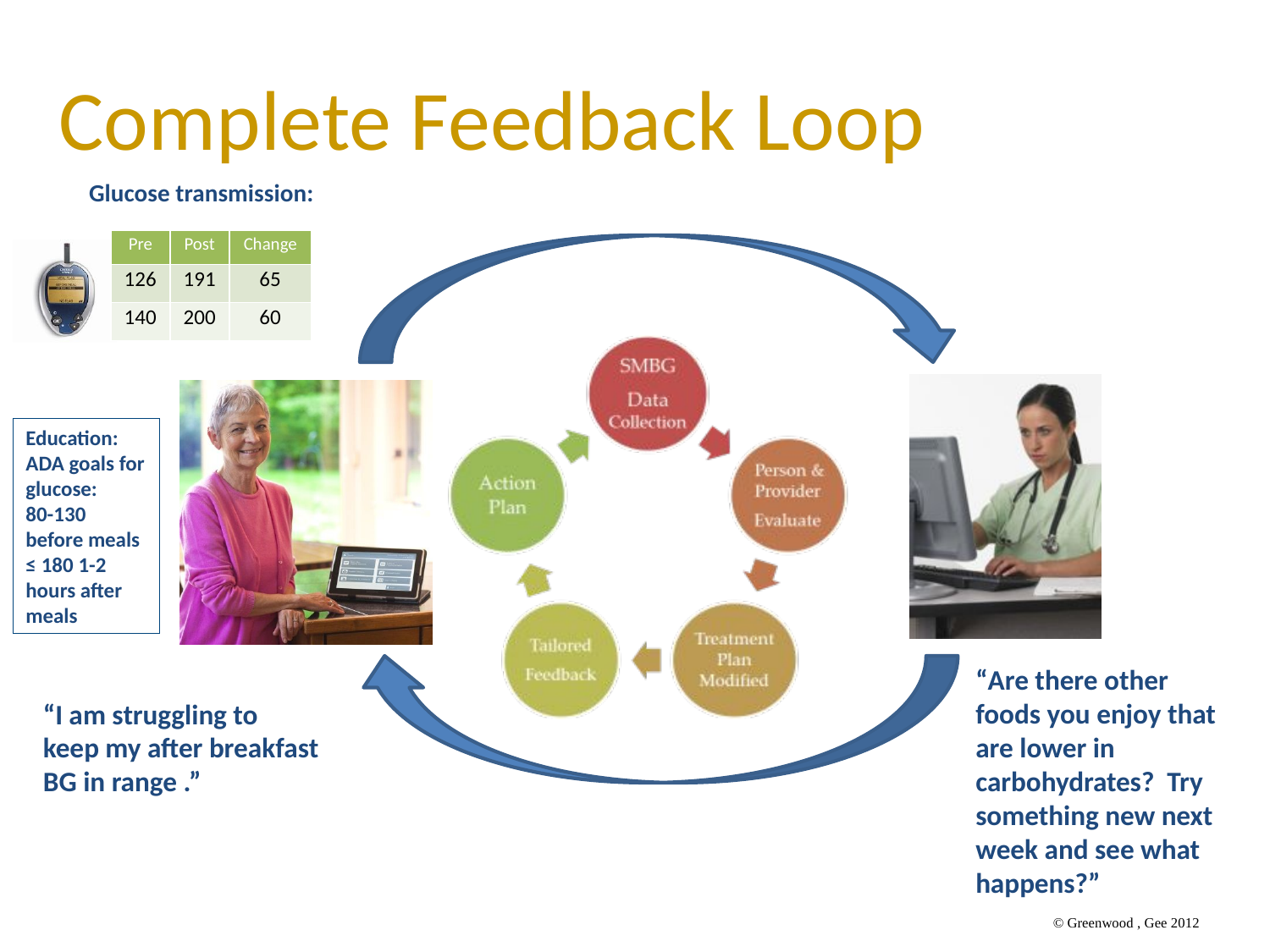

Complete Feedback Loop
Glucose transmission:
| Pre | Post | Change |
| --- | --- | --- |
| 126 | 191 | 65 |
| 140 | 200 | 60 |
Education:
ADA goals for glucose:
80-130 before meals
≤ 180 1-2 hours after meals
“Are there other foods you enjoy that are lower in carbohydrates? Try something new next week and see what happens?”
“I am struggling to keep my after breakfast BG in range .”
© Greenwood , Gee 2012

## Slide 8
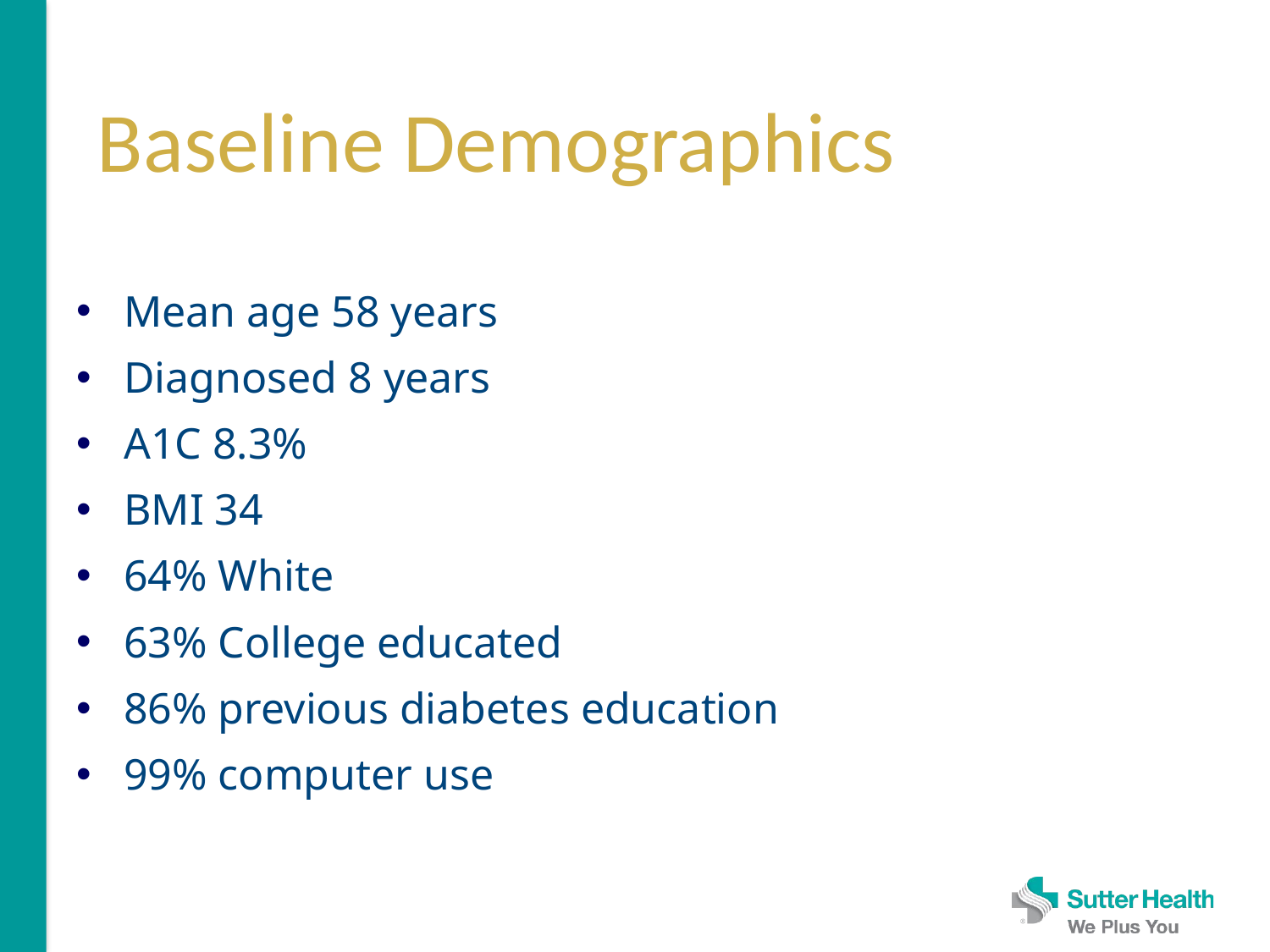

# Baseline Demographics
Mean age 58 years
Diagnosed 8 years
A1C 8.3%
BMI 34
64% White
63% College educated
86% previous diabetes education
99% computer use

## Slide 9
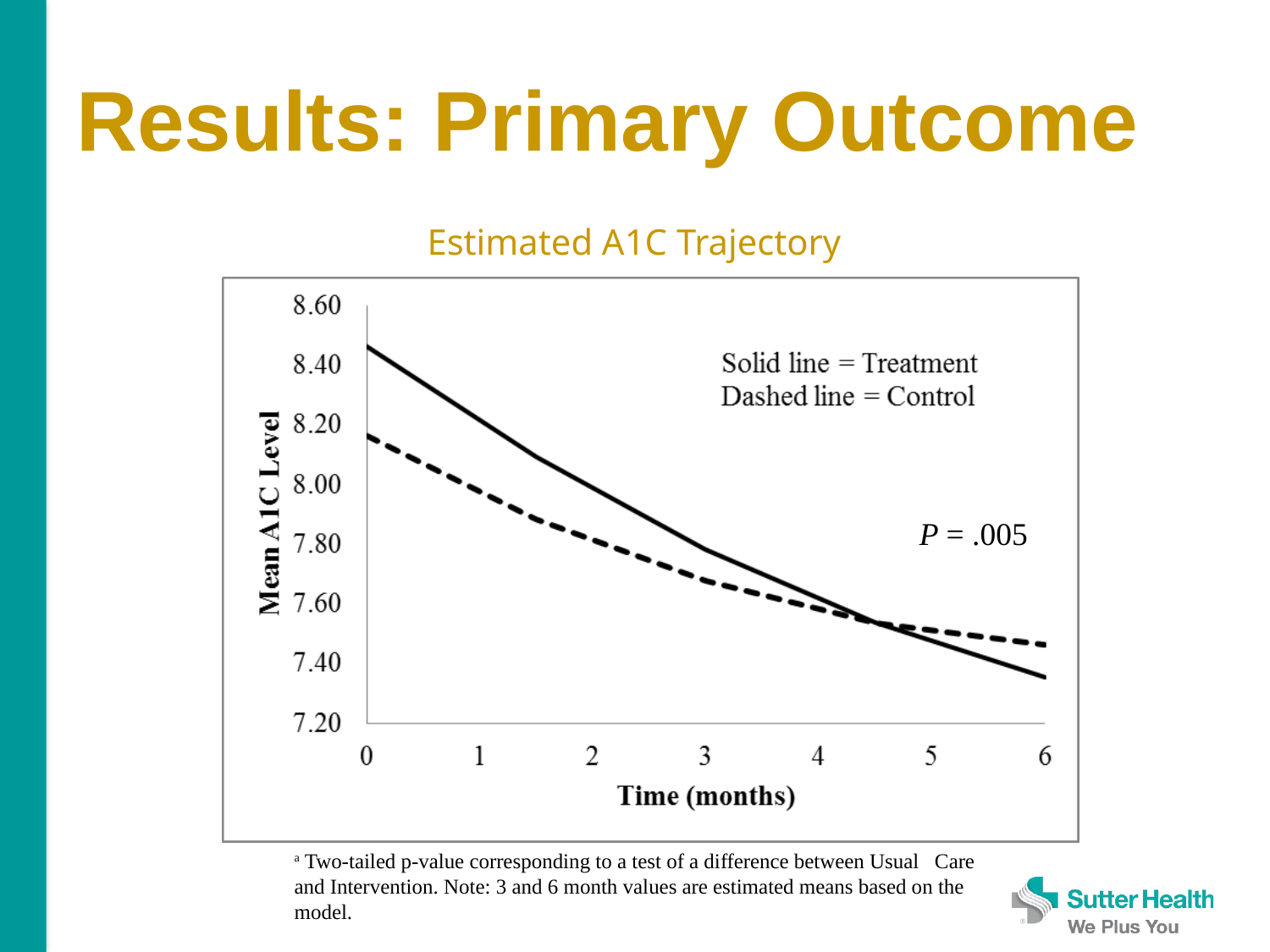

# Results: Primary Outcome
Estimated A1C Trajectory
P = .005
a Two-tailed p-value corresponding to a test of a difference between Usual Care and Intervention. Note: 3 and 6 month values are estimated means based on the model.

## Slide 10
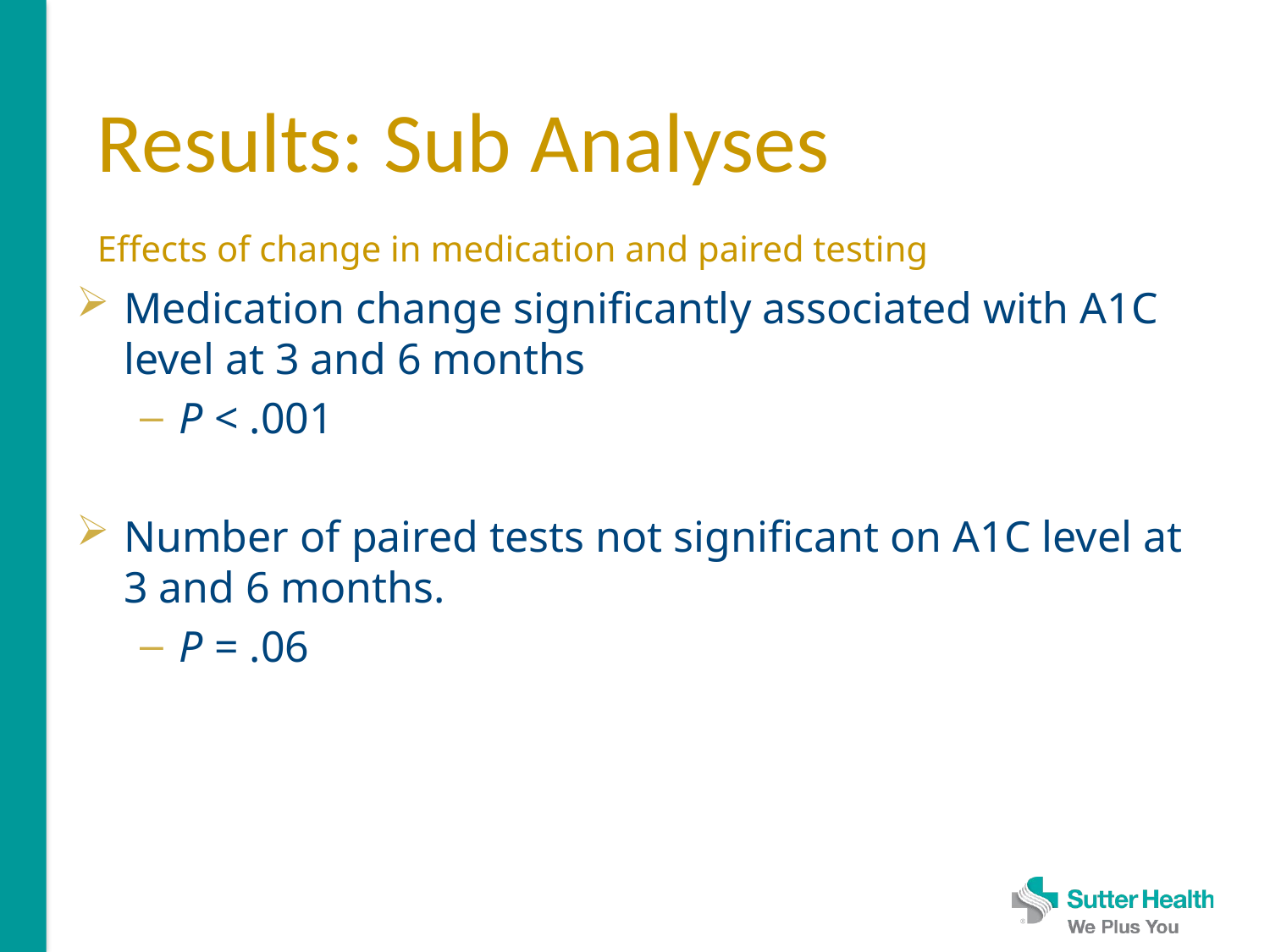

# Results: Sub Analyses
Effects of change in medication and paired testing
Medication change significantly associated with A1C level at 3 and 6 months
P < .001
Number of paired tests not significant on A1C level at 3 and 6 months.
P = .06

## Slide 11
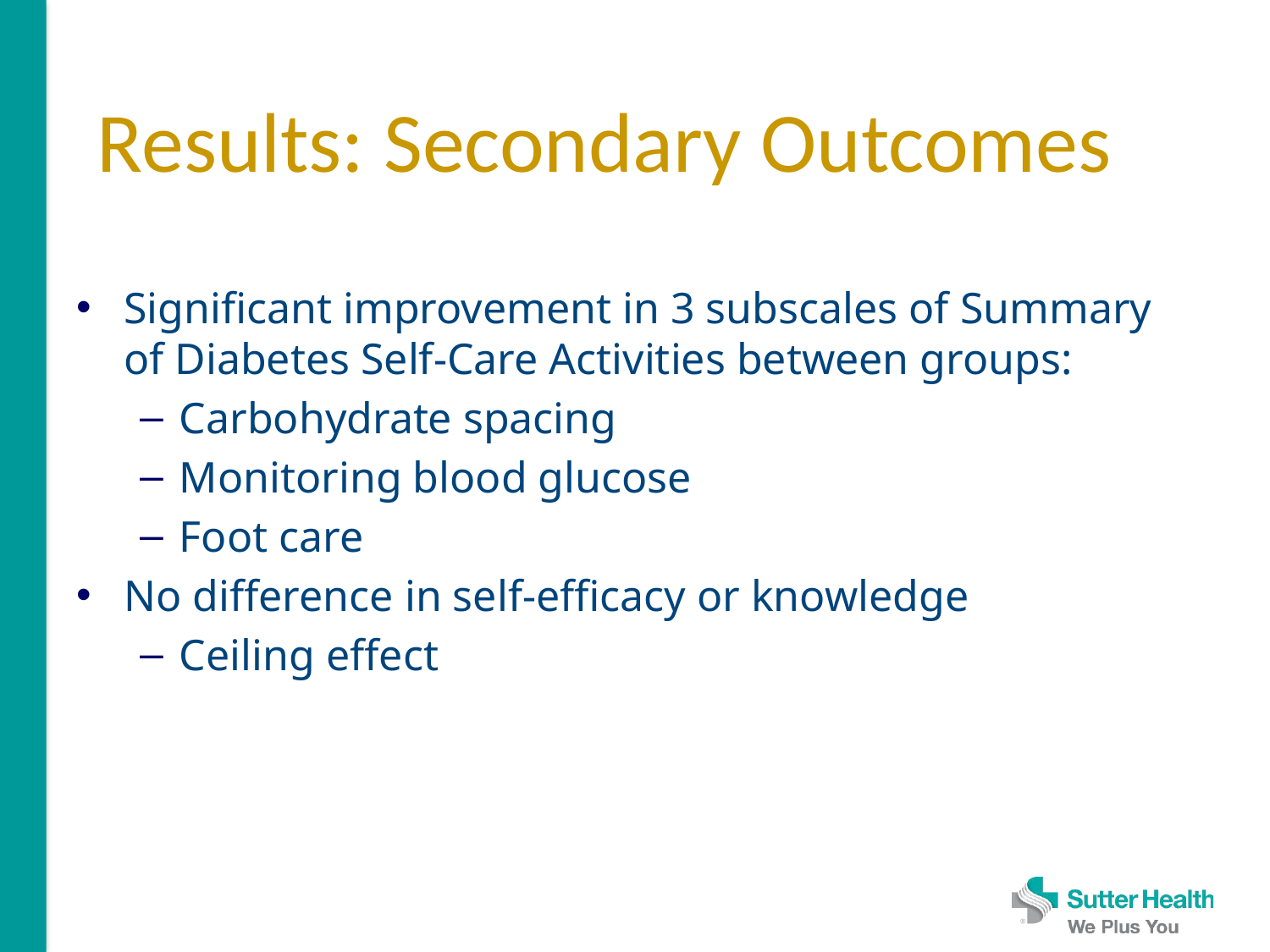

# Results: Secondary Outcomes
Significant improvement in 3 subscales of Summary of Diabetes Self-Care Activities between groups:
Carbohydrate spacing
Monitoring blood glucose
Foot care
No difference in self-efficacy or knowledge
Ceiling effect

## Slide 12
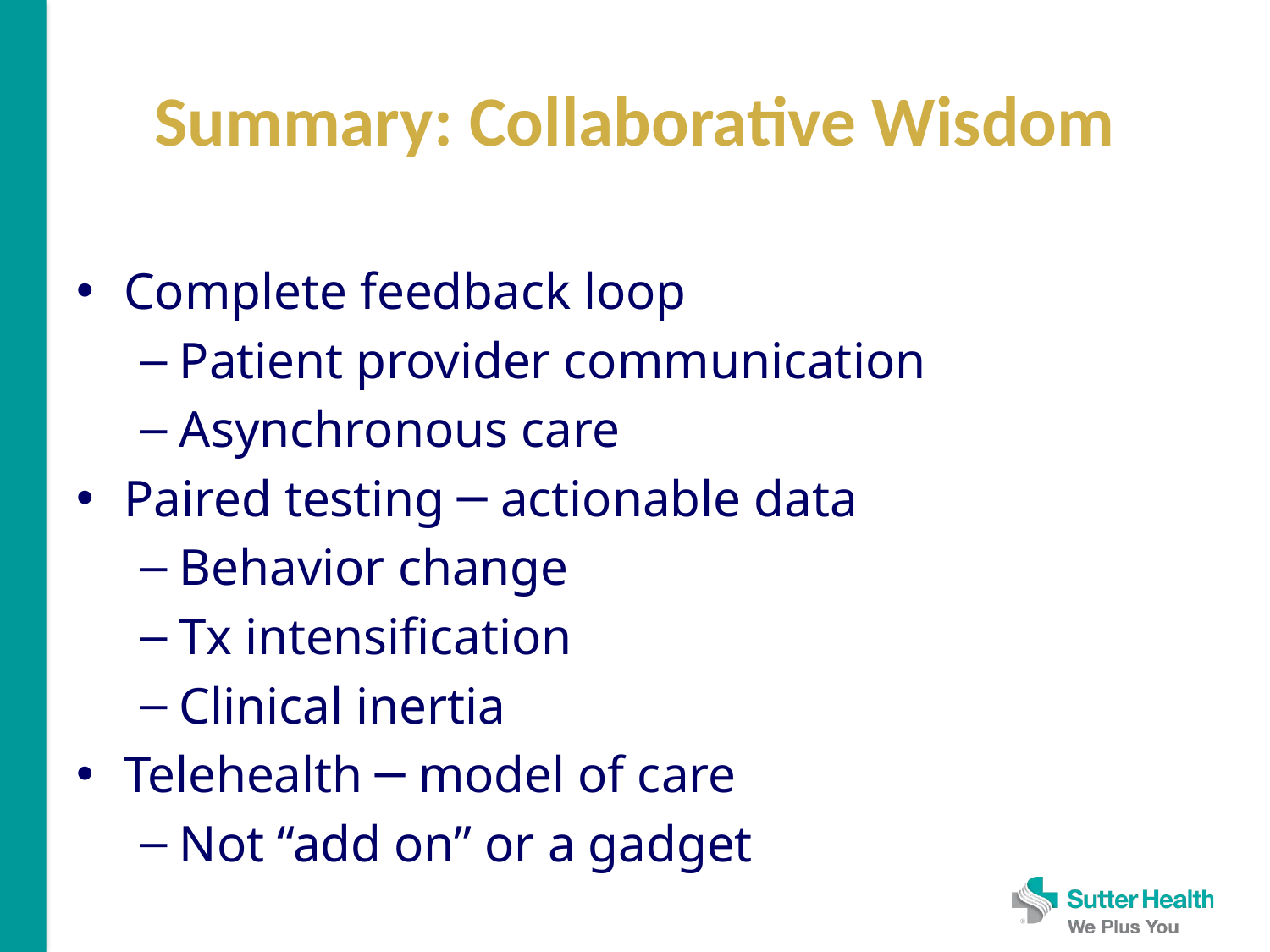

# Summary: Collaborative Wisdom
Complete feedback loop
Patient provider communication
Asynchronous care
Paired testing ─ actionable data
Behavior change
Tx intensification
Clinical inertia
Telehealth ─ model of care
Not “add on” or a gadget

## Slide 13
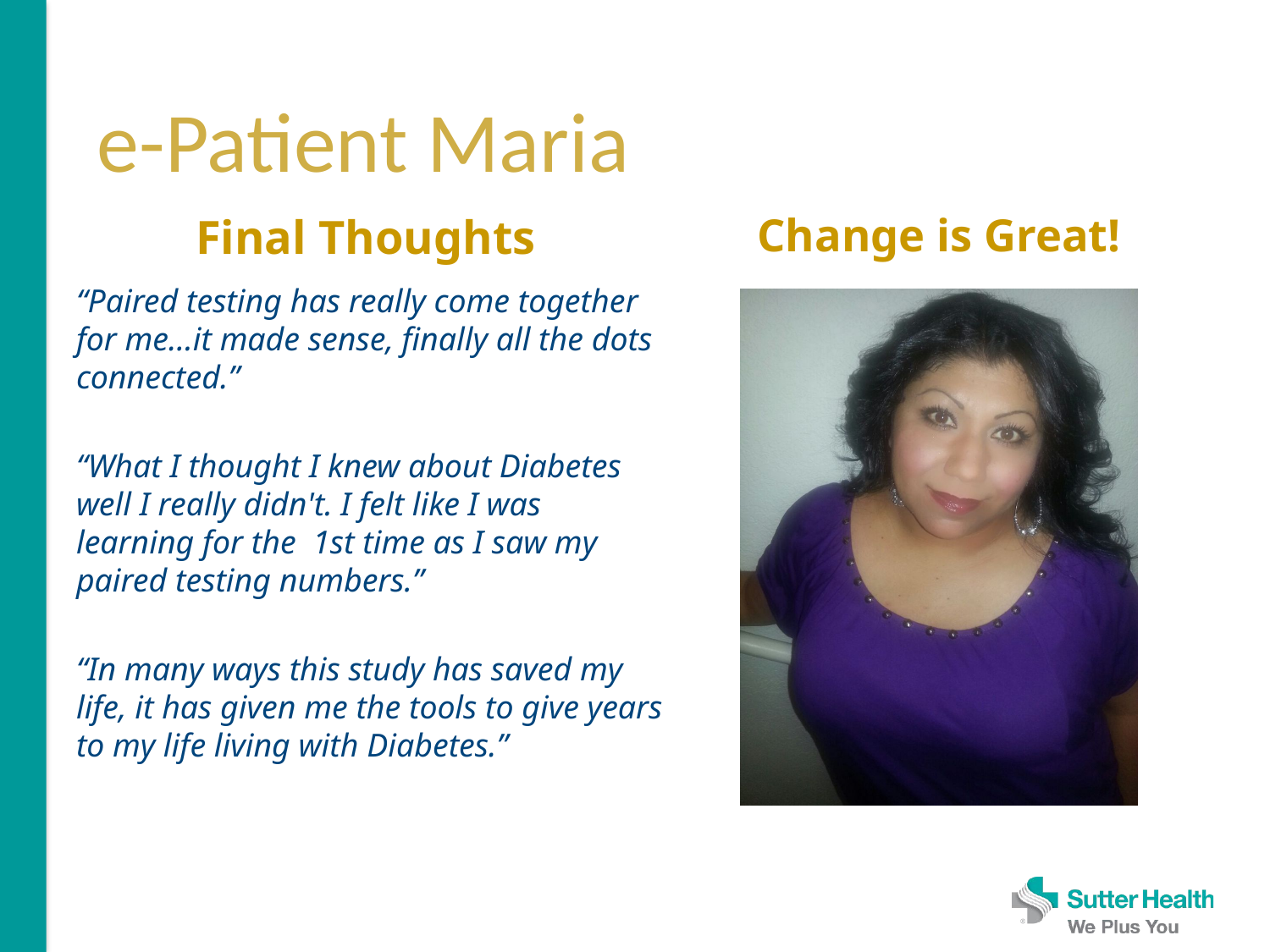

# e-Patient Maria
Change is Great!
Final Thoughts
“Paired testing has really come together for me…it made sense, finally all the dots connected.”
“What I thought I knew about Diabetes well I really didn't. I felt like I was learning for the 1st time as I saw my paired testing numbers.”
“In many ways this study has saved my life, it has given me the tools to give years to my life living with Diabetes.”

## Slide 14
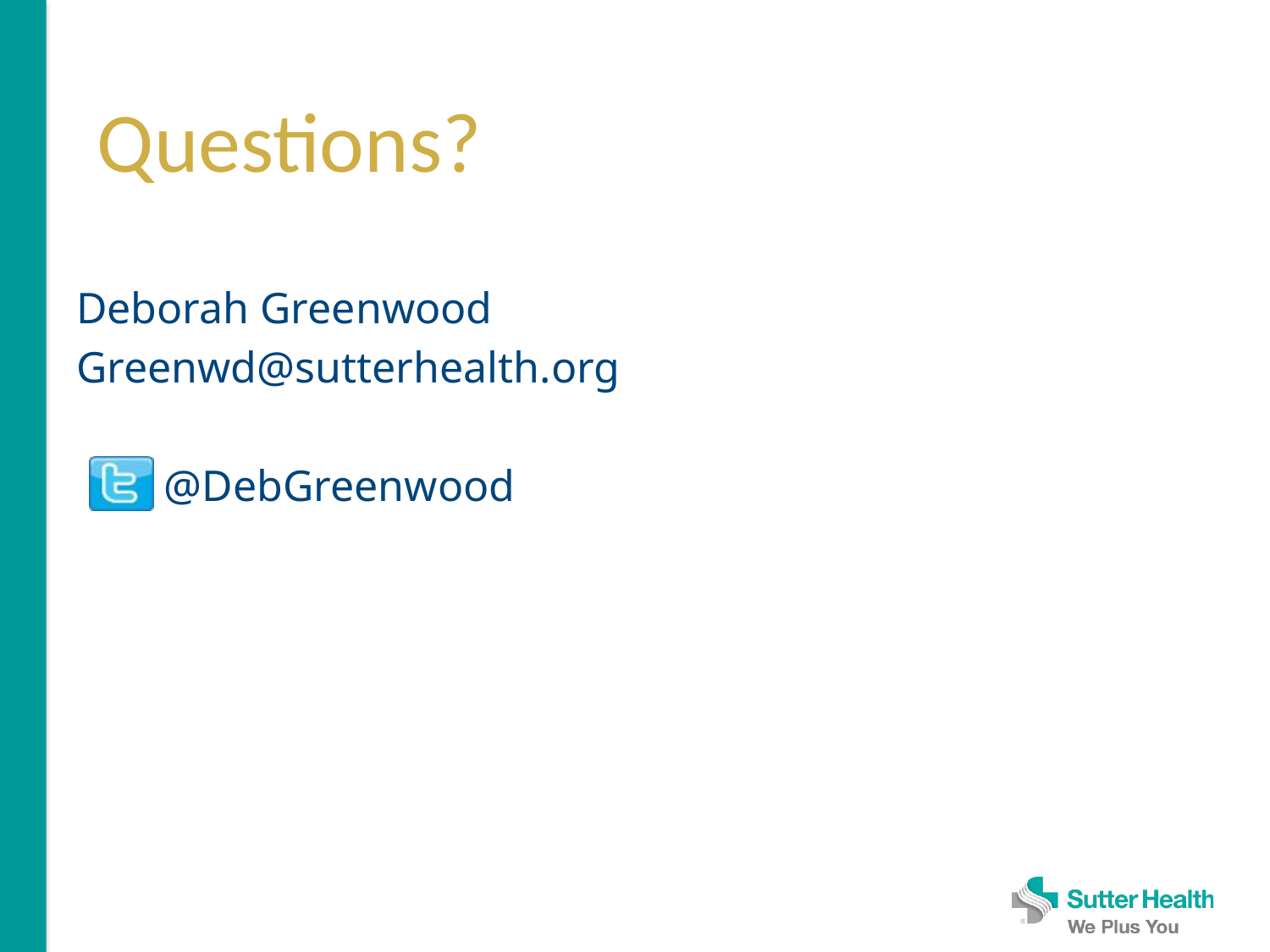

# Questions?
Deborah Greenwood
Greenwd@sutterhealth.org
 @DebGreenwood
